# SMAD1/5-mediated recruitment of the histone demethylase KDM1A controls cell fate programs in embryonic stem cells

**DOI:** 10.1016/j.jbc.2025.110591

**Published:** 2025-08-12

**Authors:** Masato Morikawa, Daizo Koinuma, Hiroaki Sakai, Yusuke Kanda, Keiko Yuki, Koji Okamoto, Kohei Miyazono

**Affiliations:** 1 Division of Health Science, Advanced Comprehensive Research Organization (ACRO), Teikyo University, Itabashi-ku, Tokyo, Japan; 2 Department of Molecular Pathology, Graduate School of Medicine, The University of Tokyo, Bunkyo-ku, Tokyo, Japan; 3 Department of Applied Pathology, Graduate School of Medicine, The University of Tokyo, Bunkyo-ku, Tokyo, Japan

**Keywords:** bone morphogenetic protein, SMAD transcription factor, histone demethylase, transcription enhancer, embryonic stem cell

## Abstract

Bone morphogenetic proteins (BMPs) play diverse roles in mouse embryonic stem cell (mESC) biology. Recent studies suggest that BMPs induce multiple cell fates and enhance mESC heterogeneity by cross-activating multiple signaling pathways. Although BMPs primarily signal through SMAD1 and SMAD5 in mESCs, their roles remain incompletely defined. Here, we investigated the SMAD signaling pathway using *Smad1/5* double knockout (S1/5 dKO) mESCs. While SMAD1/5 depletion may influence mESC heterogeneity, single-cell RNA sequencing (scRNA-seq) revealed only minor differences between S1/5 dKO and WT cells, suggesting that the observed changes are not because of altered cell states. Chromatin immunoprecipitation sequencing (ChIP-seq) demonstrated that SMAD1/5 recruit the histone demethylase KDM1A/LSD1 to specific genomic regions, where it removes H3K4me1/2 marks associated with enhancers. Published scRNA-seq data from *Kdm1a*-deficient mESCs during embryoid body differentiation further supported this mechanism. This study reveals a transcriptional repression mechanism of SMAD1/5, involving KDM1A-dependent H3K4me1/2 depletion and the regulation of cell type–specific gene expression programs.

Mouse embryonic stem cells (mESCs), derived from and expanded from the inner cell mass (ICM) of preimplantation embryos, are examples of pluripotent stem cells (PSCs) (1, 2, 3). *In vitro*, mouse PSCs can be maintained in various pluripotent states, including ground-naïve, metastable-naïve, formative, and primed pluripotent states (1, 4). These pluripotent states recapitulate the characteristics of distinct developmental stages, such as preimplantation ICM (naïve), prestreak epiblast (formative), and postimplantation epiblast (primed). Initially, culture media supplemented with serum and leukemia inhibitory factor (LIF), hereafter referred to as SL, were used to derive and maintain mESCs (1, 2, 3). In serum-containing culture conditions, mESCs exist in a heterogeneous state and fluctuate between a naïve ICM-like state and a primed epiblast-like state, known as the metastable pluripotent state. In addition, mESCs cultured in SL possess a rare transient subset (∼1%) that exhibits several features of totipotent cells in mouse zygotes and blastomeres at the two-cell (2C) stage after fertilization, referred to as two-cell-like cells (2CLCs) (5).

Bone morphogenetic protein 4 (BMP4) has been shown to replace serum and sustain the self-renewal of metastable-naïve mESCs in conjunction with LIF–signal transducer and activator of transcription 3 signaling (6) (Fig. 1*A*). BMP ligands belong to the transforming growth factor β (TGF-β) family and transmit their signals through distinct combinations of type I and type II receptors of the TGF-β family (7, 8, 9, 10) (Fig. 1*B*). Activated type I receptors phosphorylate receptor-regulated SMAD proteins (R-SMADs): SMAD1, SMAD5, and SMAD8 (encoded by the *Smad9* gene) in the BMP signaling pathway and SMAD2 and SMAD3 in the TGF-β–activin pathway. Following phosphorylation, two R-SMAD molecules form a heterotrimeric complex with SMAD4, translocate into the nucleus, and primarily bind enhancer regions to modulate gene transcription, either activating or repressing target genes. The current model proposes that the SMAD complex selects its DNA-binding target sites through cooperation with lineage-determining transcription factors (TFs) and other signal-dependent TFs (7, 11, 12), enabling context-dependent and cell type–specific biological functions. While SMAD activation is the primary mechanism governing TGF-β–BMP family signaling, the receptors also engage in additional signaling pathways, including the mitogen-activated protein kinase and PI3K–AKT–mammalian target of rapamycin pathways. These are collectively termed non-SMAD signaling pathways (13).

**Figure 1:**
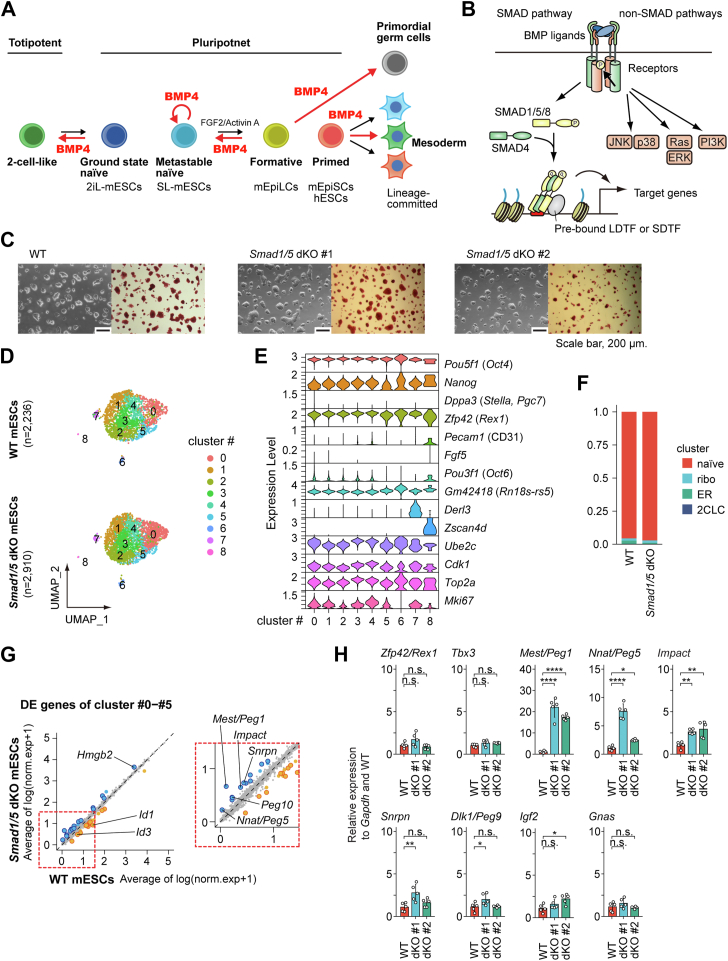
**Single-cell RNA-**s**eq analysis of *Smad1/5*-deficient mESCs and parental mESCs**. *A*, a schematic illustration of roles of BMP signaling in the biology of pluripotent stem cells (PSCs). *B*, a schematic illustration of signaling transduction of BMP signaling. *C*, morphology (*left*) and alkaline phosphatase staining (*right*) of S1/5 dKO cell lines in LIF + serum. Scale bar represents 200 μm. *D*, uniform manifold approximation and projection (UMAP) visualization of the transcriptional heterogeneity of parental E14 mESCs (WT, n = 2236) and *Smad1/5*-deficient mESCs (S1/5 dKO, n = 2910). Cells are colored based on the clusters they belong to. *E*, stacked violin plots of the integrated data showing the expression profile of marker genes: pluripotency (*Pou5f1*/OCT4), naïve pluripotency (*Nanog*, *Zfp42*), formative/primed pluripotency (*Fgf5*, *Pou3f1*/OCT6), late 2-cell stage (*Zscan4d*), and G2/M phase (*Ube2c*, *Cdk1*, *Top2a*). *F*, stacked bar plot showing the fraction of cell subtypes determined by marker gene expression. *G*, scatter plots showing expression patterns of genes in WT and S1/5 dKO cells. Average expression of log-normalized genes, difference between the transcriptomic profiles of WT and S1/5 dKO cells. Among the differentially expressed genes (DEGs), top 40 genes (20 upregulated and 20 downregulated) are highlighted in *orange* and *light blue*, respectively. In addition, those with SMAD1/5 binding within 10 kb from their transcription start sites (TSSs) are represented as *rimmed circles*. *Orange*: WT > S1/5 dKO genes, *light blue*: WT < S1/5 dKO genes. *H*, qRT–PCR analysis of mRNA of indicated genes in WT and S1/5 dKO cells. *Gapdh* was used as an endogenous control. Results of n = 5 independent biological replicates are presented as scatter plots with bar graphs, which indicate mean ± SD. Differences between the conditions were analyzed by Tukey's honestly significant difference test corrected for multiple comparisons. ∗, ∗∗, ∗∗∗, ∗∗∗∗, and ns represent *p* < 0.05, 0.01, 0.001, 0.0001, and not significant, respectively. BMP, bone morphogeneic protein; dKO, double KO; EpiLC, mouse epiblast stem cell-like cell; EpiSC, mouse epiblast stem cell; hESC, human embryonic stem cell; LDTF, lineage-determining transcription factor; LIF, leukemia inhibitory factor; mESC, mouse embryonic stem cell; P, phosphorylation; qRT–PCR, quantitative RT–PCR; SDTF, signal-dependent transcription factor.

Mouse ESCs are widely used as a model system for studying BMP signaling (14, 15) (Fig. 1*A*). BMP signaling plays a critical role in maintaining pluripotency and inhibiting neural specification by inducing SMAD1/5 target genes, such as *Id1*, and/or sustaining E-cadherin protein expression (encoded by the *Cdh1* gene) (6, 16). In addition, BMP4 has been shown to convert primed mouse epiblast stem cells into naïve mESCs (17, 18, 19). Furthermore, BMP4 has been reported to promote a transition from a pluripotent to a totipotent state, activating 2C-specific genes and increasing the percentage of 2CLCs in metastable-naïve mESCs cultured in SL (20, 21). These findings suggest that BMP signaling induces multiple cell fates and enhances ESC heterogeneity by cross-activating multiple signaling pathways (20). In contrast, modulation of BMP signaling is a critical step in many differentiation protocols (22). BMPs are potent inducers of mesodermal differentiation during embryonic development. In addition, BMP plays an essential role in the specification and expansion of primordial germ cells. These seemingly conflicting results can be explained, at least in part, by the selective use of SMAD and/or non-SMAD signaling pathways by BMP in heterogeneous metastable populations.

Since *Smad9* mRNA expression is low in mESCs, knocking out *Smad1* and *Smad5* together is sufficient to disrupt BMP–SMAD signaling. Our group and others have generated *Smad1/5*-deficient mESCs (23, 24) as well as *Smad4*-deficient mESCs (23, 25). Both models remain in a naïve pluripotent state, indicating that SMAD signaling is not essential for mESC self-renewal. However, the mechanisms underlying the diverse functions of BMPs remain incompletely defined. A key challenge in studying the SMAD signaling pathway is that its modulation can induce transitions to distinct cell states. Furthermore, SMAD1/5 exhibit distinct genomic DNA-binding profiles that are determined by cell type–specific enhancer landscapes. For example, we previously demonstrated that SMAD1/5-binding patterns in naïve mESCs differ significantly from those observed in formative epiblast stem cell–like cells (EpiLCs) (Fig. S1, *A*–*F*) (23).

The activation and silencing of enhancers are essential for regulating transcriptional programs during development and cell differentiation (reviewed in Refs. (26, 27)). Silencing or inactivation of enhancers involves mechanisms, such as DNA methylation, histone modifications (deacetylation and methylation), and various RNA-mediated processes. Lysine-specific histone demethylase 1A (KDM1A), also known as lysine-specific histone demethylase 1 (LSD1), targets enhancers to remove monomethyl and dimethyl groups from lysine 4 on histone H3 (H3K4me1/2) and plays a key role in enhancer silencing (26). Despite its role in early development, studies have shown that KDM1A is not required for the self-renewal of mESCs, as *Kdm1a*-deficient mESCs can be maintained under standard ESC culture conditions (28, 29, 30). It has been proposed that KDM1A localizes at active pluripotency enhancers in mESCs, where interaction with OCT4 suppresses KDM1A enzymatic activity (31). When mESCs are transferred to differentiation conditions, the KDM1A–nucleosome remodeling deacetylase complex removes H3K4me1 marks from pluripotency enhancers, helping to shut down the mESC gene expression program and facilitate the transition to new cell states. In addition, a recent study found that regions marked by H3K4me1 in *Kdm1a*-deficient mESCs are associated with genes related to neurogenesis (30), suggesting that KDM1A contributes to silencing the differentiation gene program in mESCs in a naïve pluripotent state.

In this study, we employed single-cell transcriptome (single-cell RNA-seq [scRNA-seq]) analysis to comprehensively compare the population characteristics of *Smad1/5*-double knockout (S1/5 dKO) mESCs established and maintained in SL (23), and parental E14 mESCs (WT). Contrary to our initial expectations, scRNA-seq revealed only minor differences between S1/5 dKO and WT cells, validating S1/5 dKO mESCs as a reliable model. Pseudobulk analysis of scRNA-seq identified differentially expressed genes (DEGs). Detailed analysis of the upregulated DEGs, genes whose expression is downregulated by SMAD1/5 (S15down genes), revealed the enrichment of monomethylation and dimethylation of H3K4me1/2 within the DEG loci of S1/5 dKO cells. Mechanistically, SMAD1/5 recruit the histone demethylase KDM1A/LSD1 to target genomic regions, facilitating the demethylation of the enhancer marks H3K4me1/2. Subsequently, we confirmed the physical interaction between SMAD1 and KDM1A. Furthermore, a custom gene set of S15down genes in the S1/5 dKO cells was enriched in *Kdm1a*-deficient cells within a subpopulation corresponding to naïve ESCs, suggesting that transcriptional repression by SMAD1/5 is dependent on KDM1A. Overall, our findings provide insights into a transcriptional repression mechanism of SMAD1/5, which may involve KDM1A-dependent enhancer silencing and the regulation of cell type–specific gene expression programs.

## Results

### Single-cell RNA-seq analysis reveals only minor differences between *Smad1/5*-deficient mESCs and parental mESCs

We previously generated genome-wide SMAD1/5-binding maps in E14 mouse ESCs (WT) under naïve SL or BMP4 + LIF conditions (Fig. 1*C*) (23). To evaluate potential changes in cell state composition upon SMAD1/5 loss, we performed scRNA-seq analysis. The data revealed largely similar distributions of pluripotent cell states between WT and S1/5 dKO cells (Figs. 1*D*, S2, *A* and *B*). We then attempted to identify marker genes in minor cell populations (Fig. 1, *D*–*F*, S2, *C*–*F*). Cluster #6 exhibited high expression of the lncRNA *Gm42418* (also known as *Rn18s-rs5*, 18s RNA-related sequence 5), which is potentially associated with ribosomal RNA contamination. Cluster #7 contained marker genes associated with endoplasmic reticulum–associated protein degradation, such as *Derl3* and *Hspa5*. In addition, cluster #8 harbored elevated expressions of 2CLC marker genes, such as *Zscan4d* and *Tcstv3*. Although we replicated that BMP4 increased the percentage of 2CLCs in mESCs cultured in SL (Fig. S2, *F* and *G*), SMAD1/5 depletion did not affect the number of cells in cluster #8 (Figs. 1*F* and S2*C*). This suggests that the SMAD pathway has minimal involvement in the induction of 2CLCs (20, 21).

We then focused on the marker genes of the predominant population (clusters #0−#5). Several well-established cell cycle markers, such as *Ube2c*, *Cdk1*, *Mki67*, and *Top2a* in the G2/M phase, were overrepresented in clusters #3 and #4 (Figs. 1*E* and S2*D*), suggesting that differences in the cell cycle status contributed to cell-to-cell heterogeneity. We then employed a tricycle algorithm for cell cycle prediction (32), which revealed no obvious difference between WT and S1/5 dKO cells in terms of the cell cycle phase (Fig. S3, *A* and *B*). This finding is consistent with previous reports based on cell proliferation assays of *Smad1/5*-deficient mESCs (23, 24).

### SMAD1/5-binding regions at the gene loci of the genes downregulated by SMAD1/5 tend to lack H3K4me1 marks

We then examined the DEGs within clusters #0 to #5. Marker genes specific to a naïve pluripotent state were not differentially expressed between WT and S1/5 dKO cells (Fig. S3, *C* and *D*). In contrast, the mRNA expression of BMP–SMAD target genes, such as *Id1* or *Id3*, decreased in the S1/5 dKO cells (Figs. 1*G* and S3*D*), validating the effects of *Smad1/5* KO. DEGs between WT and S1/5 dKO cells were screened using the FindMarkers function of Seurat (Wilcoxon rank sum test), which detected 866 DEGs with a *p* < 0.001 (Table S1). From these, top 40 genes (20 upregulated and 20 downregulated) were identified through pseudo-bulk analysis and scatter plot visualization (Fig. 1*G* and Table S2) and selected for downstream analysis. Interestingly, the mRNA expression of several paternally expressed genes (PEGs) (33), such as *Mest* (also known as *Peg1*), *Nnat* (*Peg5*), and *Impact*, increased in the S1/5 dKO cells. We performed quantitative RT–PCR (qRT–PCR) analysis and validated the upregulation of these genes using two different S1/5 dKO lines (23) (Fig. 1*H*). In contrast, the expression levels of other imprinted genes were less affected by SMAD1/5 loss: *Dlk1* (*Peg9*) and *Igf2* were upregulated in only one of the two dKO lines, and *Gnas* showed no change.

These regions overlap with the reported differentially methylated regions (DMRs) of these imprinted genes. Bisulfite sequencing revealed that the DMRs of *Mest*, *Nnat*, and *Impact* were hypermethylated in WT cells but became hypomethylated in S1/5 dKO cells. In contrast, the DNA methylation status of the *Dlk1–Meg3* intergenic DMR was only minimally affected in S1/5 dKO cells (Fig. S4*A*), suggesting that the loss of methylation was not a global event. Consistent with this, qRT–PCR analysis showed that the expression levels of *Dnmt1*, *Dnmt3a*, and *Uhrf1* were unchanged between WT and S1/5 dKO cells. In contrast, *Dnmt3b* and *Dnmt3l* showed a slight but statistically significant increase in both dKO lines (Fig. S4*B*). However, since these enzymes promote DNA methylation, their upregulation does not account for the hypomethylation observed in S1/5 dKO cells.

**Figure 2:**
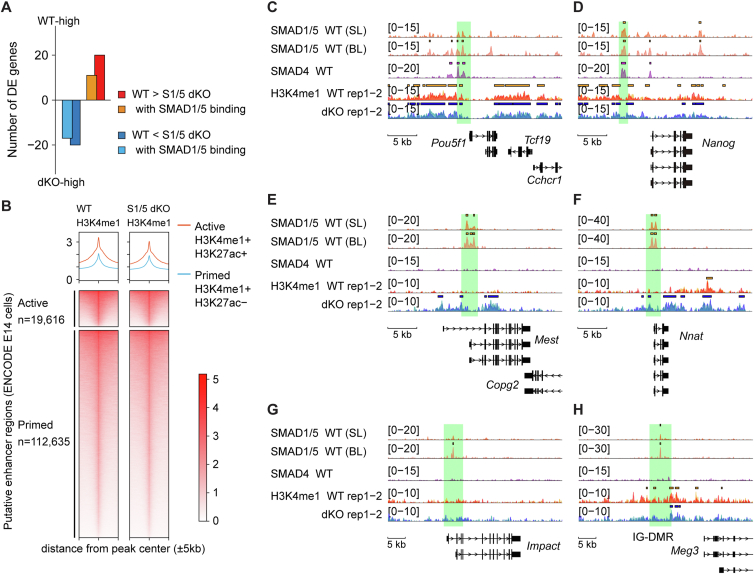
**H3K4me1 enrichment in the SMAD1/5-binding regions in naïve ESCs**. *A*, bar graph showing the number of DEGs presented in Figure 1*G*. *B*, heatmap visualization of H3K4me1 ChIP-seq data obtained with WT and S1/5 dKO cells on the enhancer regions defined in ENCODE E14 cells within a 10-kb window around the peak. *C*−*H*, genome browser tracks of the indicated normalized ChIP-seq signals at the gene loci of the genes downregulated by SMAD1/5 (S15down genes) and control genes of WT and S1/5 dKO cells. The read counts were first normalized to 1× genome coverage (reads per genome coverage, RPGC) and then normalized to input. Each ChIP-seq data are presented in a different color. *Solid bars* above ChIP peaks represent called peaks. ChIP-seq, chromatin immunoprecipitation sequencing; DEG, differentially expressed gene; dKO, double KO; ESC, embryonic stem cell; H3K4me1, monomethylation of histone H3 lysine 4.

Notably, among the 40 DEGs, 29 (72.5%) had SMAD1/5-binding sites within 10 kb of the transcription start sites (Fig. 2*A*). SMAD1/5 binding was more frequent at the gene loci of the DEGs with higher mRNA expression in S1/5 dKO (genes whose expression is downregulated by SMAD1/5, hereafter referred to as “S15down genes”; Fig. 2*A*). Since SMAD1/5 often bind enhancer regions marked with monomethylation of H3K4me1, we performed ChIP-seq analysis of H3K4me1 marks in WT and S1/5 dKO cells (Fig. 2, *B*−*H*). H3K4me1 data from the WT and S1/5 dKO cells showed comparable enrichment within the putative enhancer regions defined using the ENCODE datasets of E14 cells on a genome-wide scale (Fig. 2*B*). Furthermore, SMAD1/5 and SMAD4 co-occupied regions marked by H3K4me1 in WT and S1/5 dKO cells at the positive control loci *Pou5f1* and *Nanog* (Fig. 2, *C* and *D*; (34)). However, SMAD1/5-binding regions tended to lack H3K4me1 enrichment in WT cells, whereas SMAD1/5 loss led to increased H3K4me1 marks at the loci of S15down genes (Fig. 2, *E*−*G*). Notably, these regions did not show SMAD4 binding, suggesting that this mechanism is independent of SMAD4.

### SMAD1/5 colocalize with KDM1A/LSD1, which represses a subset of SMAD1/5 target genes

**Figure 3:**
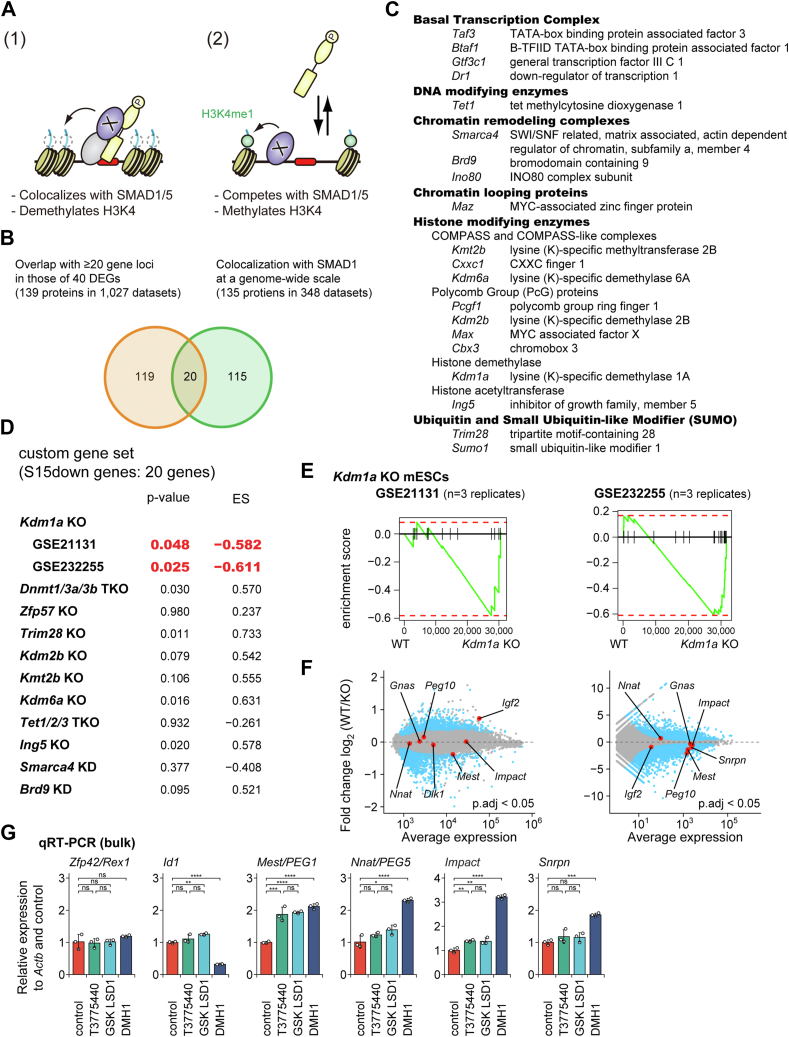
**Involvement of KDM1A/LSD1 in regulation of the genes downregulated by SMAD1/5**. *A*, a schematic illustration of candidate models governing the transcriptional regulation of S15down genes (1). SMAD1/5 colocalize with factor X that demethylates H3K4 or (2) SMAD1/5 competes with factor X that methylates H3K4. *B*, a Venn diagram indicating the overlap of proteins preferentially bind to the gene loci of 40 DEGs (1027 datasets or 139 proteins) and proteins colocalized with SMAD1 on a genome-wide scale (482 datasets or 135 proteins). *C*, a list of 20 proteins depicted within the overlapped region in Figure 3*B*. *D* and *E*, results of preranked gene set enrichment analysis (GSEA) with the custom gene set of the S15down genes (20 genes) in mESCs deficient in indicated gene(s) compared with control cells. *D*, a table presents the *p* value and enrichment score (ES) of GSEA. A negative ES indicates that members of the gene set tend to appear at the bottom of the ranked transcriptome data or highly expressed in mESCs deficient in indicated gene(s). *E*, GSEA plots showing the enrichment results of the custom gene set in *Kdm1a*-deficient mESCs of two different studies. *F*, MA plots of DESeq2 data of *Kdm1a*-deficient mESCs, showing log2 fold change (M, on the *y*-axis) against the mean of the normalized counts (A, on the *x*-axis). *Circles* colored *light blue* indicate genes whose transcript abundance were significantly altered (adjusted *p* < 0.05) based on both DESeq2. *G*, qRT–PCR analysis of mRNA of indicated genes in mESCs treated with KDM1A/LSD1 inhibitors, DMH1, or DMSO as control. *Actb* was used as endogenous control. Results of n = 3 independent biological replicates are presented as scatter plots with bar graphs, which indicate mean ± SD. Differences between the conditions were analyzed by Tukey's honestly significant difference test corrected for multiple comparisons. ∗, ∗∗, ∗∗∗, ∗∗∗∗, and ns represent *p* < 0.05, 0.01, 0.001, 0.0001, and not significant, respectively. DEG, differentially expressed gene; DMSO, dimethyl sulfoxide; mESC, mouse embryonic stem cell; qRT–PCR, quantitative RT–PCR.

Since SMAD1/5 proteins do not function as epigenetic effectors, we tried to find an SMAD cofactor (referred to as "factor X"). Specifically, we hypothesized that (1) SMAD1/5 colocalize with factor X, which demethylates H3K4, or (2) SMAD1/5 compete with factor X, which methylates H3K4 (Fig. 3*A*). To obtain clues supporting our hypotheses, we analyzed the ChIP-seq data available from the ChIP-Atlas database (35, 36). We identified 139 proteins/1027 ChIP-seq datasets out of a total of 7312 datasets that overlapped with 20 or more gene loci in the 40 DEGs discovered in this study (Figs. 3*B* and S5*A*). We also identified 135 proteins/482 datasets that colocalized with SMAD1 on a genome-wide scale. Consequently, we identified 20 proteins that could bind to the gene loci of the S15down genes and colocalize with SMAD1 (Fig. 3, *B* and *C*).

We also utilized publicly available transcriptomic data from KO mESCs deficient in the 20 candidate genes listed in Figure 3*C*. In addition to the candidate genes, we also evaluated ZFP57, a Krüppel-associated box (KRAB)–containing zinc finger protein, and DNA methyltransferases (DNMTs), which are involved in maintaining DNA methylation of imprinted genes during early embryonic stages together with TRIM28 (33). We focused on KDM1A because the custom gene set derived from the S15down genes (20 genes) was enriched in *Kdm1a*-deficient mESCs in two different studies (GSE21131 and GSE232255; Fig. 3*D*, 3*E*, and S5*B)*. In addition, the loss of KDM1A/LSD1 increased the mRNA expression of *Mest* and several S15down genes in naïve mESCs (Fig. 3*F*). Treatment with two different chemical inhibitors of KDM1A/LSD1 consistently increased the mRNA expression of *Mest* and several S15down genes in WT mESCs (Fig. 3*G*).

### SMAD1 physically interacts with KDM1A through the Mad homology 2 domain of SMAD1

**Figure 4:**
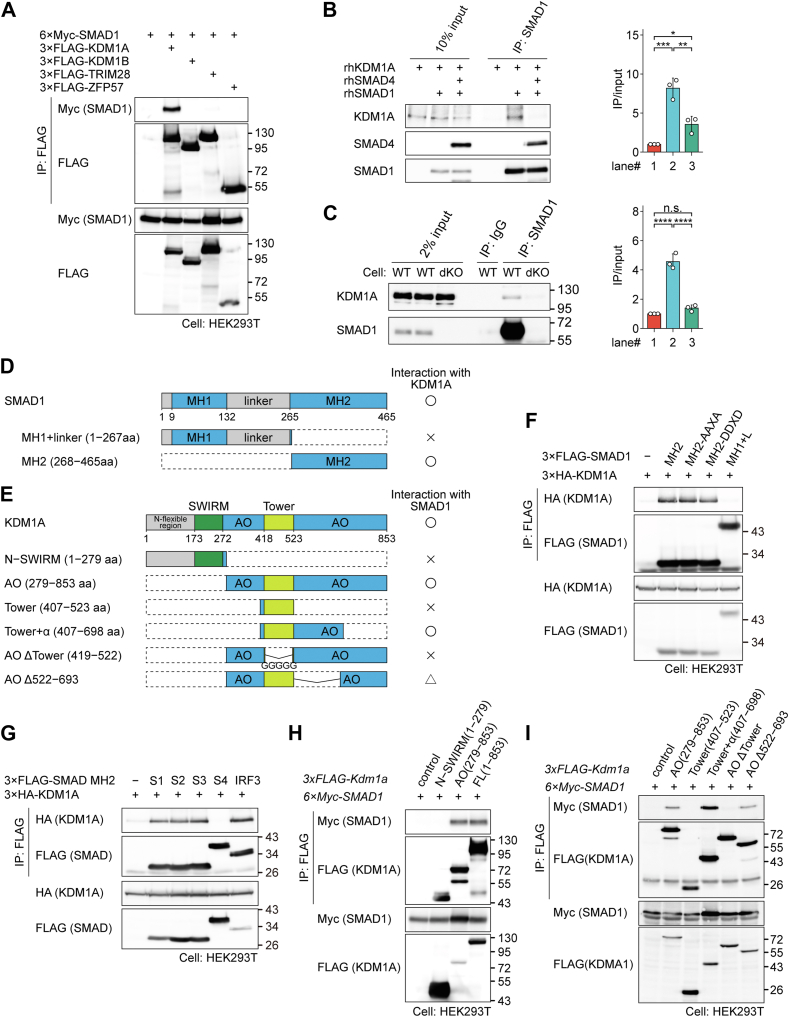
**Physical interaction between SMAD1 and KDM1A**. *A*, coimmunoprecipitation of Myc-tagged SMAD1 with the indicated FLAG-tagged coregulators in HEK293T cells. FLAG immunoprecipitates were subjected to Western blotting. Blots are representative of n = 3 independent biological replicates. *B*, *in vitro* pulldown of KDM1A (0.5 μg) and/or SMAD4 (0.5 μg) with SMAD1 (0.5 μg) as bait. Blots are representative of n = 3 independent biological replicates. *Right*, quantification of band intensity is shown on the *right*. Results of n = 3 independent biological replicates are presented as scatter plots with bar graphs, which indicate mean ± SD. Differences between the conditions were analyzed by Tukey's honestly significant difference test corrected for multiple comparisons. *C*, coimmunoprecipitation of endogenous SMAD1/5 protein with KDM1A in WT mESCs. SMAD1/5 immunoprecipitates were subjected to Western blotting. Blots are representative of n = 3 independent biological replicates. *Right*, quantification of each lane of the blots. Results of n = 3 independent biological replicates are presented as scatter plots with bar graphs, which indicate mean ± SD. Differences between the conditions were analyzed by Tukey's honestly significant difference test corrected for multiple comparisons. *D* and *E*, schematic illustrations of interaction between SMAD1 and KDM1A. *F*, coimmunoprecipitation of HA-tagged KDM1A with the indicated FLAG-tagged SMAD1 mutants in HEK293T cells. FLAG immunoprecipitates were subjected to Western blotting. Blots are representative of n = 3 independent biological replicates. *G*, coimmunoprecipitation of HA-tagged KDM1A with the indicated FLAG-tagged MH2 domain of SMAD protein or IAD of IRF3 in HEK293T cells. FLAG immunoprecipitates were subjected to Western blotting. Blots are representative of n = 3 independent biological replicates. *H*, coimmunoprecipitation of Myc-tagged SMAD1 with the indicated FLAG-tagged KDM1A mutants in HEK293T cells. FLAG immunoprecipitates were subjected to Western blotting. Blots are representative of n = 3 independent biological replicates. *I*, coimmunoprecipitation of Myc-tagged SMAD1 with the indicated FLAG-tagged KDM1A mutants in HEK293T cells. FLAG immunoprecipitates were subjected to Western blotting. Blots are representative of n = 3 independent biological replicates. ∗, ∗∗, ∗∗∗∗, and ns represent *p* < 0.05, 0.01, 0.0001, and not significant, respectively. HEK293T, human embryonic kidney 293T cell line; mESC, mouse embryonic stem cell; MH2, Mad Homology 2 domain.

The colocalization observed in ChIP-seq does not necessarily confirm a physical interaction between colocalized proteins. To determine whether SMAD1/5 physically interacts with KDM1A, we conducted an *in vitro* analyses. SMAD1 and SMAD5 proteins exhibit interchangeable roles in early mouse embryogenesis (37) and share conserved amino acid sequences; therefore, SMAD1 was selected for immunoprecipitation (IP) experiments. In human embryonic kidney 293T (HEK293T) cells, KDM1A interacted with SMAD1, whereas KDM1B (previously known as LSD2/AOF1), TRIM28, and ZFP57 did not (Fig. 4*A*). This interaction was further validated *in vitro* using recombinant proteins (Fig. 4*B*). Intriguingly, recombinant SMAD4 protein disrupted the interactions between SMAD1 and KDM1A. We also confirmed this interaction using endogenous proteins in mESCs *in vivo* (Fig. 4*C*). Notably, KDM1A was not immunoprecipitated in the S1/5 dKO cells (Fig. 4*C*). These results suggest that KDM1A interacts with the Mad homology 2 (MH2) domain of SMAD1. The MH2 domain is also essential for forming a complex between SMAD1 and SMAD4. Domain mapping experiments confirmed that the MH2 domain of SMAD1 is required for its interaction with KDM1A (Fig. 4, *D*−*F*). Interestingly, a phosphomimetic mutant, SMAD1 MH2 DDXD, exhibited binding affinity similar to that of the phosphorylation-resistant mutant, SMAD1 MH2 AAXA (Fig. 4*F*). The amino acid sequence of the MH2 domain is conserved among R-SMAD proteins (Fig. S6). In addition, the interferon-activating domain (IAD) of IRF3 shares significant structural and surface electrostatic similarity with the MH2 domains (38). Similar to SMAD1 MH2, the MH2 domains of SMAD2, SMAD3, and IRF3 IAD interacted with KDM1A. In contrast, the MH2 domain of SMAD4 did not interact with KDM1A (Fig. 4*G*). Finally, we mapped the binding domain of KDM1A and identified the "tower domain" as necessary, but not sufficient, for its interaction with SMAD1 (Fig. 4, *E*, *H* and *I*).

### SMAD1/5 recruit KDM1A to the target sites and demethylate H3K4me1/2

**Figure 5:**
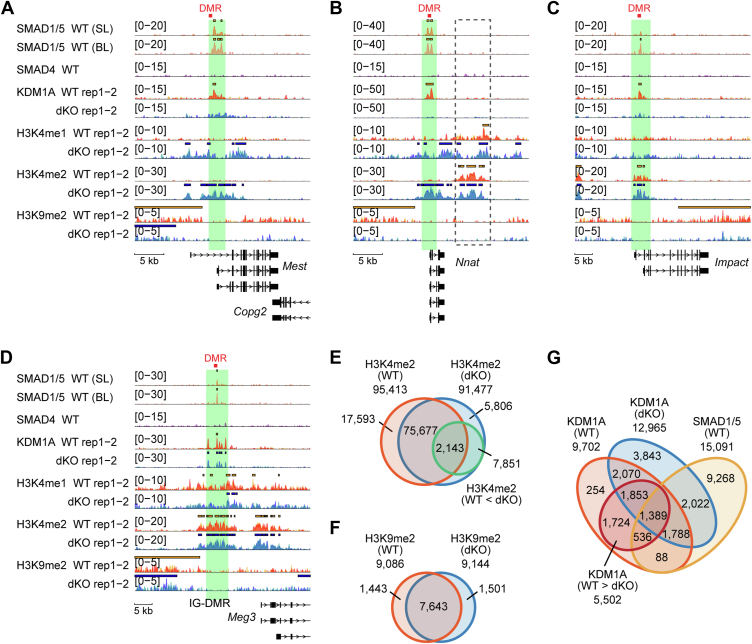
**Evaluation of H3K4me2 and H3K9me2 at SMAD1/5-binding regions in naïve ESCs**. *A*–*D*, genome browser tracks of the indicated normalized ChIP-seq signals at the gene loci of SMAD1/5-repressed genes and a control gene of WT and S1/5 dKO cells. The read counts were first normalized to 1× genome coverage (reads per genome coverage, RPGC) and then normalized to input. Each ChIP-seq data are presented in a different color. *Solid bars* above ChIP peaks represent called peaks. DMR, PCR-amplified regions used for the bisulfite sequencing analysis in Figure S4*A*. *E*–*G*, Venn diagrams indicating the overlap of indicated genomic regions identified using ChIP-seq data. *E*, ChIP-seq data of H3K4me2 in either WT cells or S1/5 dKO cells and H3K4me2 WT < S1/5 dKO cells (1.5-fold). *F*, ChIP-seq data of H3K9me2 in either WT cells or S1/5 dKO cells. *G*, ChIP-seq data of KDM1A in either WT cells or S1/5 dKO cells, SMAD1/5 and KDM1A WT > S1/5 dKO cells (1.5-fold). ChIP-seq, chromatin immunoprecipitation sequencing; dKO, double KO; ESC, embryonic stem cell.

KDM1A/LSD1 is a histone demethylase that specifically removes monomethyl and dimethyl groups from lysine 4 or lysine 9 of histone 3 (H3K4 or H3K9, respectively) (26). To investigate how the SMAD1/5–KDM1A complex affects histone methylation, we analyzed ChIP-seq data of H3K4me2 and H3K9me2 in WT and S1/5 dKO cells (Fig. 5, *A*−*D*). In WT cells, the shared binding regions of SMAD1/5 and KDM1A, such as the gene loci of *Mest* and *Nnat*, exhibited only subtle modifications in H3K4me2. However, in S1/5 dKO cells, where KDM1A binding was lost, these regions showed an enrichment of H3K4me2. In contrast, H3K9me2 was not strongly affected by SMAD1/5 deletion at the same gene loci. Interestingly, another H3K4me2-positive region located 5 kb from the SMAD1/5-binding region within the *Nnat* locus was preserved in the S1/5 dKO cells (Fig. 5*B*, *black dashed line area*). In addition, genome-wide patterns of H3K4me2 and H3K9me2 showed minimal alterations (Fig. 5, *E* and *F*). We then identified 5502 regions with a ≥1.5-fold reduction in KDM1A levels in S1/5 dKO cells compared with WT cells (Fig. 5*G*). Of these, 1925 regions (1389 + 536, 35.0%) colocalized with SMAD1/5-binding regions in WT cells (Fig. 5*G*).

**Figure 6:**
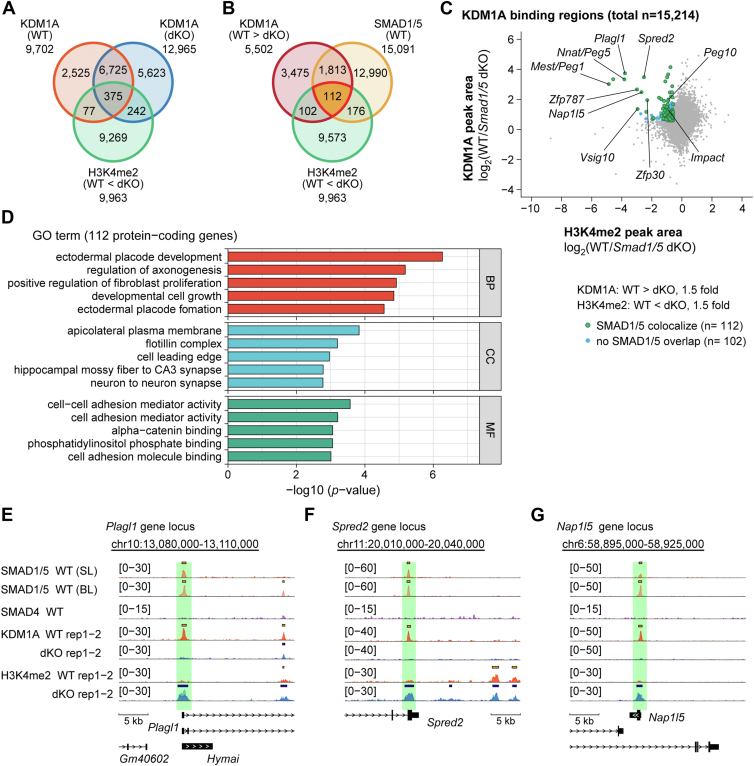
**SMAD1/5-dependent recruitment of KDM1A and demethylation of H3K4me1/2 innaïve ESCs**. *A* and *B*, Venn diagrams indicating the overlap of indicated genomic regions identified using ChIP-seq data. *A*, ChIP-seq data of KDM1A in either WT cells or S1/5 dKO cells and H3K4me2 WT < S1/5 dKO cells (1.5-fold), (*B*) SMAD1/5, KDM1A in WT > S1/5 dKO cells (1.5-fold), H3K4me2 in WT < S1/5 dKO cells (1.5-fold). The overlapped area indicates 112 genomic regions. *C*, scatter plot illustrating the relationship between KDM1A binding and H3K4me2. *D*, over representation analysis (ORA) for Gene Ontology (GO) terms. Protein-coding genes associated (112) with the 112 genomic regions. *E*−*G*, examples of SMAD1/5-binding regions with decreased KDM1A binding and increased demethylation of H3K4me2 in S1/5 dKO cells. Genome browser tracks of the indicated normalized ChIP-seq signals at the gene loci of indicated genes of WT and S1/5 dKO cells were presented. The read counts were first normalized to 1× genome coverage (reads per genome coverage, RPGC) and then normalized to input. Each ChIP-seq data are presented in a different color. *Solid bars* above ChIP peaks represent called peaks. ChIP-seq, chromatin immunoprecipitation sequencing; dKO, double KO; ESC, embryonic stem cell.

We then assessed whether SMAD1/5 facilitated KDM1A recruitment and mediated the demethylation of H3K4me1/2 on a genome-wide scale. In S1/5 dKO cells, 9963 regions displayed a ≥1.5-fold increase in H3K4me2 levels compared with WT cells (Fig. 6, *A* and *B*). Of these, only 694 (77 + 375 + 242) were linked to KDM1A binding (Figs. 6*A*), and 214 (102 + 112) regions showed a ≥1.5-fold reduction in KDM1A binding after SMAD1/5 loss (Fig. 6*B*). We focused on the 214 regions with decreased KDM1A binding and increased H3K4me2 methylation in the S1/5 dKO cells (Fig. 6*B*), where KDM1A likely functions as an H3K4me2 demethylase. Of these 214 regions, 112 (52.3%) colocalized with SMAD1/5-binding sites in WT mESCs (Fig. 6, *B* and *C*). The relatively small size of this subset suggests that KDM1A’s demethylase activity is restricted to a limited number of loci, rather than being broadly active across its binding sites. One possible explanation for this is the inhibition of KDM1A's histone demethylation activity by OCT4, as reported previously (31). Gene Ontology (GO) analysis of these 112 regions suggested their association with developmental processes toward several lineages, including the neuroectoderm (NE) and the preplacode region at the border of the neural plate (Fig. 6*D*). Representative genome browser views of several gene loci are presented in Figures 6, *E*−*G* and S7, *A*−F, whereas those of control regions are shown in Figure S8, *A*−*D*. These findings suggest that SMAD1/5 play a pivotal role in recruiting KDM1A to its target sites, and the complex represses a subset of SMAD1/5 target genes.

### SMAD1/5–KDM1A target regions in mESCs correspond to loci enriched for H3K4me2 in EpiLCs

To examine how SMAD1/5–KDM1A-dependent demethylation relates to chromatin state changes during early differentiation, we performed SMAD1/5 ChIP-seq in both naïve mESCs and formative/primed mEpiLCs (Figs. S9–S11). We focused on the subset of 112 genomic regions, defined as loci where SMAD1/5 facilitate KDM1A recruitment and H3K4me2 removal (Fig. 6, *B* and *C*). As a control, we used 10,678 SMAD1/5-bound regions that lack H3K4me2 in WT mESCs (Fig. S11*D*). Only 7.0% of these control regions overlapped with H3K4me2 peaks in mEpiLCs, whereas 70.5% of the 112 SMAD1/5–KDM1A candidate regions showed such overlap (Fig. S11*E*). These findings suggest that SMAD1/5–KDM1A-dependent demethylation helps maintain the regions in an H3K4me1/2-negative state in naïve mESCs, and that loss of this demethylation may lead to H3K4me1/2 accumulation during the transition to formative pluripotency.

### KDM1A suppresses PSC differentiation to specific lineages

**Figure 7:**
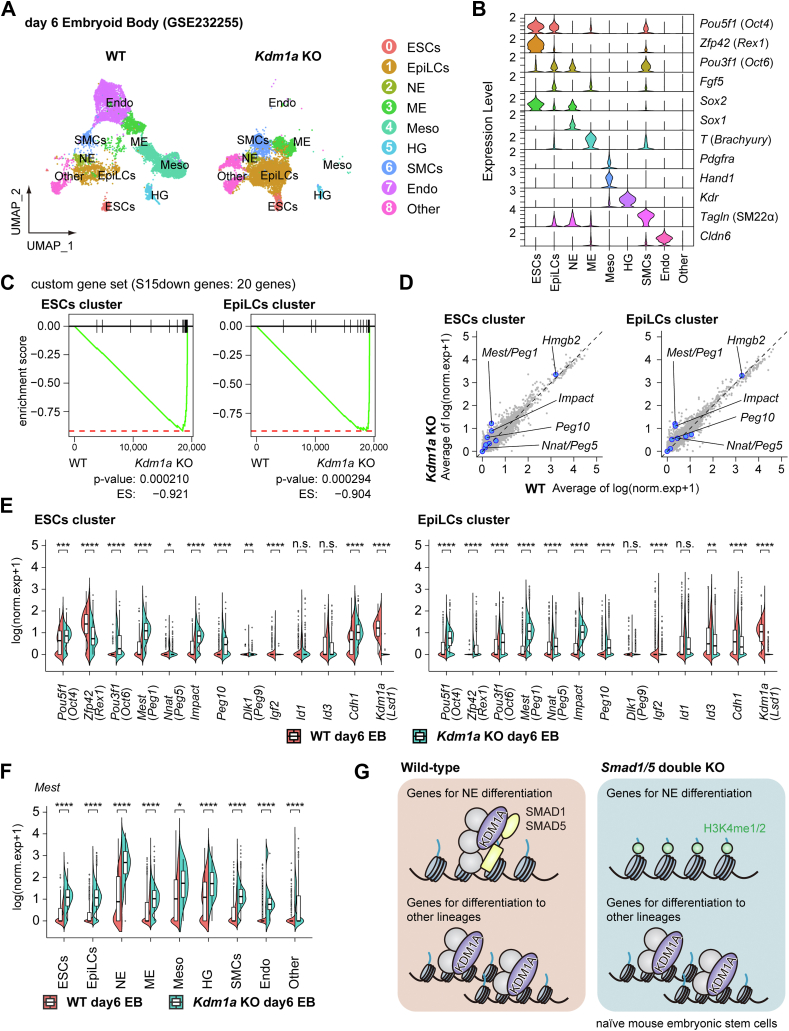
**Effects of KDM1A loss in PSC differentiation to specific lineages**. *A*, UMAP plot showing the cell types present in the day 6 embryoid body generated from *Kdm1a*-deficient mESCs (n = 7623) and control (n = 10,214) (29). Cells are colored based on the clusters they belong to. *B*, stacked violin plots of the integrated data showing the expression profile of marker genes: pluripotency (*Pou5f1*/OCT4), naïve pluripotency (*Zfp42*), formative/primed pluripotency (*Pou3f1*/OCT6, *Fgf5*), NE (*Sox2*, *Sox1*), ME (*T*), Meso (*Pdgfra*, *Hand1*), HG (*Kdr*), SMCs (*Tagln*), and Endo (*Cldn6*). *C*, GSEA plots showing the enrichment results of the custom gene set of the S15down genes (20 genes) in *Kdm1a*-deficient cells within subpopulations corresponding to naïve ESCs and formative/primed EpiLCs. *D*, scatter plots showing expression patterns of genes in *Kdm1a*-deficient cells within subpopulations corresponding to naïve ESCs and formative/primed EpiLCs. Average expression of log-normalized genes, difference between the transcriptomic profiles of WT and *Kdm1a* KO. *E* and *F*, half-violin plots showing (*E*) the distribution of the mRNA expression levels of indicated genes in either WT or *Kdm1a*-deficient cells within the subpopulations corresponding to naïve ESCs and formative/primed EpiLCs, and (*F*) the distribution of *Mest* mRNA expression level in either WT or *Kdm1a*-deficient cells within the indicated subpopulations. Each cell was treated as an independent replicate, and differences in gene expression between conditions were analyzed using the Wilcoxon rank sum test. *G*, model for the roles of the SMAD1/5–KDM1A complex in metastable-naïve mouse ESCs cultured in serum plus LIF (SL). SMAD1/5 recruit KDM1A to the target sites and demethylate H3K4me1/2. The SMAD1/5–KDM1A complex, which does not contain SMAD4, represses a subset of SMAD1/5 target genes related to developmental processes toward several lineages, including neuroectoderm (NE). cDNA, complementary DNA; DMR, differentially methylated region; DNMT1, DNA methyltransferase 1; EB, embryoid body; Endo, endoderm; ES, enrichment score; GEO, Gene Expression Omnibus; GO, Gene Ontology; HG, *Flk1*^*+*^ hemangioblast; IAD, interferon-activating domain; IP, immunoprecipitation; LSD1, lysine-specific histone demethylase 1; mAG, monomeric Azami-Green; ME, mesendoderm; Meso, mesoderm; MH2, Mad Homology 2 domain; NE, neuroectoderm (*Sox1*^+^ NE progenitor); NM, nascent mesoderm; PEG, paternally expressed gene; qRT–PCR, quantitative RT–PCR; SMC, smooth muscle cell; TF, transcription factor; TGF-β, transforming growth factor β; VE, visceral endoderm.

To further evaluate the biological significance of SMAD1/5-dependent recruitment of KDM1A, we focused on a differentiation model of *Kdm1a*-deficient mESCs. scRNA-seq data from day 6 embryoid bodies (EBs) generated using *Kdm1a*-deficient mESCs revealed that *Kdm1a* deletion led to a reduction in the number of mesodermal and endodermal cells within the EBs (Fig. 7, *A* and *B*, and S12*A*), as described in a previous study (29). Notably, the custom gene set of the S15down genes in the S1/5 dKO cells (20 genes) was enriched in *Kdm1a*-deficient cells within subpopulations corresponding to naïve ESCs and formative/primed EpiLCs (Fig. 7, *C* and *D*). In addition, the mRNA expression of *Mest* and several S15down genes increased in *Kdm1a*-deficient cells in both subpopulations (Fig. 7, *E*, *F*, and S12*B*). Interestingly, we found the enrichment of *Mest* and *Nnat* transcripts in subpopulations corresponding to *Sox1*^+^ NE progenitors in WT cells (Figs. 7*F* and S12*B*). Although the transcriptomic changes in S1/5 dKO cells are similar to those observed in *Kdm1a*-deficient mESCs, the latter exhibit a greater number of DEGs, reflecting additional KDM1A functions that are independent of SMAD1/5. Therefore, this study reveals a transcriptional repression mechanism of SMAD1/5, involving KDM1A-dependent enhancer silencing and the regulation of cell type–specific gene expression programs.

## Discussion

Several studies, including the recently reported work by Lee *et al*. (39) focus on enhancer activation, where repressive histone marks are removed by SMAD-containing complexes; however, the role of SMAD-mediated enhancer silencing remains underexplored. Our findings demonstrate that SMAD1/5 recruit KDM1A to specific genomic sites, leading to the demethylation of H3K4me1/2 and repression of a subset of SMAD1/5 target genes (Fig. 6, *C* and *D* and 7*G*). The change in H3K4me1/2 levels is not a secondary effect of shifts in cell-type composition following SMAD1/5 loss, as confirmed by scRNA-seq. GO analysis indicated that the silenced enhancer regions were associated with NE differentiation (Fig. 6*D*). The mRNA expression of *Mest*, one representative gene in this context, increased during EB differentiation, whereas the mRNA expression of *Nestin*, one of early lineage markers, was significantly reduced in *Mest*-deficient cells (40). Furthermore, *Nnat* inactivation led to a failure of neural induction during EB formation, even under BMP pathway inhibition (41). These findings are consistent with the well-established role of BMP as an antineural factor in vertebrate embryos (6, 16).

Our ChIP-seq data validated that SMAD1/5 recruit KDM1A to specific genomic sites (Figs. 5 and 6). KDM1A is a critical chromatin regulator that catalyzes the removal of H3K4me1/2 (42), and DNA-binding factors that tether KDM1A to specific genomic sites are essential for its function (26). Several TFs have been shown to recruit KDM1A to its target sites. Proteins containing SNAG domains, such as GFI1, GFI1B (43), and SNAI1 (also known as SNAIL) (44) serve as molecular hooks for recruiting KDM1A/LSD1. The interaction was mapped to the MH2 domain of SMAD1, which is well conserved among SMAD1/5/8 and SMAD2/3 (SMAD2/3 mediate TGF-β/activin signaling) (Fig. S6). Interactions between KDM1A and either SMAD8 (45) or SMAD2/3 (46) have been previously described. These observations suggest that SMAD2/3 may also recruit KDM1A to their target sites. Interestingly, SMAD2/3 have been implicated in suppressing the expression of extraembryonic genes during priming and differentiation (47). They employ the nucleosome remodeling deacetylase–containing complex in conjunction with transcriptional enhanced associate domain to repress mesodermal gene enhancers, leading to transcriptional repression in human ESCs (48). Furthermore, we identified an interaction between KDM1A and the IAD of IRF3 (Fig. 4*G*), which shares structural similarities with the MH2 domains. This finding adds a layer of complexity to SMAD–KDM1A-mediated enhancer regulation, as activated IRF3 could disrupt the formation of SMAD–KDM1A complexes and their biological responses, similar to what has been observed in R-SMAD–SMAD4 complexes (49, 50).

We confirmed that SMAD1 interacts with KDM1A in an SMAD4-independent manner (Fig. 4*B*). Although SMAD4 is a key component of the SMAD complex, SMAD4-independent R-SMAD-mediated biological responses have been documented (10). For example, C-terminally unphosphorylated SMAD3 has been shown to enter the nucleus and exert biological effects (51). In addition, SMAD2/3 have been reported to regulate specific transcriptional programs in an SMAD4-independent manner, possibly through interacting with TRIM33 (also known as TIFγ or ectodermin) (52) or NKX2-1 (TTF-1) (53). In the current study, TRIM28 (also known as TIFβ), a TRIM33 family member, was identified as an SMAD1 colocalizing factor (Fig. 3*C*). However, we did not detect any physical interaction between TRIM28 and SMAD1/5 under our experimental conditions (Fig. 4*A*).

The relationship between KDM1A/LSD1 and DNA methylation status has been extensively documented. KDM1A is known to demethylate various substrates, including histone proteins and nonhistone proteins (reviewed in Refs. (26, 27)). One of the nonhistone targets of KDM1A is DNMT1 (54) and its cofactor UHRF1, an E3 ubiquitin protein ligase (55). By demethylating and stabilizing DNMT1 and/or UHRF1, KDM1A influences global DNA methylation patterns in mESCs. Another mechanism linking KDM1A and DNA methylation involves the DNMT3A–DNMT3L complex. DNMT3L is a cofactor with no methyltransferase activity that forms a complex with the *de novo* methyltransferase DNMT3A (56, 57). KDM1A-mediated histone demethylation facilitates interactions between H3K4me0 and the DNMT3A–DNMT3L complex, thereby promoting *de novo* DNA methylation at specific loci (58). In this study, we observed local, rather than global, DNA hypomethylation in S1/5 dKO cells (Fig. S4*A*), whereas mRNA expression of *Dnmt3b* and *Dnmt3l* was slightly elevated in S1/5 dKO cells (Fig. S4*B*). Therefore, the loss of SMAD1/5-mediated KDM1A recruitment in S1/5 dKO cells may have caused hypomethylation at regions bound by SMAD1/5 in WT cells. This site-specific hypomethylation may be linked to the recruitment of the DNMT3A–DNMT3L complex, as the absence of H3K4 methylation does not prevent DNMT3L binding.

In conclusion, this study provides insights into the molecular mechanism of transcriptional repression by SMAD1/5, which is mediated by the SMAD4-independent recruitment of KDM1A. Although SMAD-mediated transcriptional repression has been previously described (7, 9), the role of SMAD1/5 in the enhancer silencing of target genes remains underexplored. Our findings demonstrate that the SMAD1/5–KDM1A complex represses genes related to developmental processes toward several lineages, including the NE, in naïve mESCs (Figs. 7*G* and S12*C*). Further studies are needed to investigate the function of the SMAD–KDM1A complex in various biological contexts, such as tumorigenesis and epithelial-to-mesenchymal transition, processes in which BMPs and other TGF-β family members play critical roles.

### Limitations of the study

Our study has several limitations. First, mESCs maintained under static conditions were used. In the case of retinoic acid, KDM1A/LSD1-mediated enhancer silencing regulates the duration of the transcriptional response, forming a feedback loop during pancreatic endocrine cell development (59). Although the C-terminal phosphorylation of SMAD is not required, the SMAD1/5–KDM1A complex may predominantly function in later phases following BMP treatment, potentially contributing to the formation of a negative feedback loop. Second, because of the relatively weak binding affinity of the DNA-binding MH1 domain, the SMAD complex likely cooperates with high-affinity DNA-binding TFs to locate target sites. Additional DNA-binding TFs, such as SNAI1, which is expressed in naïve mESCs (60), may be required to accurately identify SMAD1/5 target genes associated with developmental processes leading to NE. Consequently, the precise mechanism by which SMAD1/5 recognizes target loci lacking H3K4me1 remains unclear and should be addressed in future investigations.

## Experimental procedures

### Cell culture

Feeder-free E14 mouse ESCs were maintained under SL conditions containing serum, and *Smad1*^−/−^;*Smad5*^−/−^ double KO mESCs (S1/5 dKO) was generated using CRISPR–Cas9 system (23). These cells were maintained as reported with slight modification (23); cells were cultured at 37 °C in 5% CO_2_ and were maintained on gelatin (Millipore, Merck, or FUJIFILM Wako)-coated dishes in Dulbecco's modified Eagle's medium (Gibco, Thermo Fisher Scientific), with 15% embryonic stem cell–qualified fetal bovine serum (Millipore, Merck), 0.1 mM β-mercaptoethanol (FUJIFILM Wako), 2 mM L-glutamine, 1× MEM nonessential amino acids, 100 U/ml penicillin and 100 μg/ml streptomycin (all from Gibco), and 1000 U/ml LIF (Millipore).

HEK293T cells were obtained from the American Type Culture Collection. Lenti-X 293T cells were purchased from Clontech (TaKaRa Bio). HEK293T and lenti-X 293T cells were maintained in Dulbecco's modified Eagle's medium (Gibco), supplemented with 10% (v/v) fetal bovine serum (HyClone, GE Healthcare Life Sciences/Cytiva) and 100 U/ml penicillin–streptomycin (Gibco).

### Reagents and antibodies

Recombinant human SMAD1 (ab84653; Abcam) and human SMAD4 (ab81764; Abcam) were purchased from Abcam. Recombinant N-terminal FLAG-tagged human KDM1A (#31426; Active Motif) was purchased from Active Motif.

Antibodies against the following proteins were used: mouse monoclonal anti-SMAD1 (913C1b; Bio Matrix Research), which recognizes both SMAD1 and SMAD5 (61) for IP and ChIP assays, rabbit polyclonal anti-SMAD1 (#9743; Cell Signaling) for Western blotting, mouse monoclonal anti-SMAD4 (sc-7966; Santa Cruz), anti-KDM1A (ab17721; lot #GR307810-1, Abcam) for ChIP, anti-KDM1A (sc-271720; Santa Cruz Biotechnology) for WB, anti-FLAG M2 (F3165; Sigma–Aldrich, Merck), anti-Myc (9E10; FUJIFILM Wako), anti-HA (HA124; Nacalai Tesque), anti-H3K4me1 (#07-436, lot #3120573; Millipore), anti-H3K4me2 (#07-030, lot #3506381; Millipore), and anti-H3K9me2 (ab1220, lot #GR212253-11; Abcam) for ChIP-seq.

The following low-molecular weight kinase inhibitors were used: LSD1 inhibitors GSK-LSD1 and T-3775440 were purchased from Sigma–Aldrich/Merck and Selleck Chemicals, respectively. BMP type I receptor inhibitor DMH1 was also obtained from Selleck Chemicals.

### Alkaline phosphatase staining

Alkaline phosphatase staining was performed using Leukocyte Alkaline Phosphatase Kit (Sigma–Aldrich, Merck), following the manufacturer's protocol.

### Plasmid construction

The coding region of mouse *Kdm1a* (GeneBank accession: NM_133872.2), *Kdm1b* (NM_172262.3), *Trim28* (NM_011588.3), *Zfp57* (NM_001013745.2), and human *IRF3* (NM_001571.6) were amplified by PCR and were subcloned into the pcDEF3-5′3×FLAG, pcDNA3-5′3×FLAG, pcDNA3-5′6×Myc, pcDNA3-5′3×HA, pcDNA3-3′3×FLAG, and pcDNA3-3′3×HA vectors (62). The constructs of SMAD1, SMAD2, SMAD3, and SMAD4 were previously described (23, 61). Deletion mutants were generated using PCR with specific primers with sites for restriction enzymes with stop codon. Details of mutants are as follows. SMAD1 MH1+linker (L): 1 to 267 aa (amino acids), SMAD1 MH2: 268 to 465 aa, SMAD2 MH2: 271 to 467 aa, SMAD3 MH2: 229 to 425 aa, SMAD4 MH2: 320 to 552 aa, IRF3 IAD: 200 to 427 aa, KDM1A N-SWIRM: 1 to 267 aa, KDM1A amine oxidase: 279 to 853 aa, KDM1A Tower: 407 to 523 aa, KDM1A Tower+α: 407 to 698 aa, KDM1A AOΔtower: 419 to 522 aa, and KDM1A AOΔ522 to 693 (Fig. 4, *D* and *E*). We introduced point mutations at the C terminus of the MH2 domains using site-directed mutagenesis with specific primers.

The CSI-6×BRE(GC)-monomeric Azami-Green (mAG)-Neo contains 6-time 25-nt minimal BMP Responsive Element and super core promoter (23, 63). The construct was generated using CSI-3×BRE(GA)-AcGFP-Hyg and pGL4-3×BRE(GC)-DsRed-Neo (23). CSI-MERVL-tdTomato-Hyg was generated using 730 nucleotides promoter sequence of *MERVL* (5) and CSI-3×GA-AcGFP-Hyg (23). All the constructs for lentivirus production were kindly provided by Dr H. Miyoshi (deceased; formerly RIKEN). All the constructs were verified by Sanger sequencing.

### Lentivirus production

Recombinant lentiviruses were produced by transient transfection using Lipofectamine 2000 (Invitrogen, Thermo Fisher Scientific) in HEK293T or Lenti-X 293T cells and were concentrated using the Lenti-X Concentrator (Clontech, TaKaRa Bio).

### Bisulfite Sanger sequencing

Genomic DNA was isolated using a Gentra Puregene Cell kit (Qiagen, Thermo Fisher Scientific). DNA bisulfite conversion was performed using Epitect Bisulfite kits (Qiagen). The targeted regions were amplified with TaKaRa Epi-Taq HS (for bisulfite-treated DNA) (TaKaRa Bio). Primer sets used are listed in Table S3. The PCR for *Mest*, *Nnat*, *Impact*, and *Dlk1-Meg3* IG was done as follows: 95 °C for 2 min; repeat steps 40×; 95 °C for 10 s; 55 °C for 30 s; 72 °C for 30 s; hold 16 °C. The resulting amplified products were gel-purified, subcloned into pCR2.1-TOPO vector (Thermo Fisher Scientific), and sequenced using M13 reverse primer. Results were analyzed for change in methylation pattern with the QUMA software (64).

### RNA isolation, qRT–PCR

Different cell lines (WT, S1/5 dKO #1, and #2) were stably maintained, and total RNA was extracted at various time points, which may have differed between cell lines. For inhibitor treatments, cells were cultured for more than 7 days in SL medium supplemented with either 1 μM T3775440, 2 μM GSK-LSD1, 1 μM DMH1, or dimethyl sulfoxide (vehicle control). Total RNA from mESCs was prepared using an RNeasy Mini Kit (Qiagen) following the manufacturer's protocol. First-strand complementary DNA (cDNA) was synthesized using a PrimeScript II first strand cDNA Synthesis Kit (Takara Bio). qRT–PCR was performed in a StepOnePlus Real-Time PCR Systems (Applied Biosystems, Thermo Fisher Scientific) using FastStart Universal SYBR Green Master Mix with Rox (Roche, Merck). Relative quantification was performed using the ΔΔCt method, and the data were normalized with *Gapdh*, or *Actb*, depending on the condition. LSD1 inhibitors were found to influence metabolic genes, leading to variability in *Gapdh* expression. Primer sequences are given in Table S4.

### ChIP-seq and data analysis

Chromatin isolation, sonication, and IP using antibody were performed essentially as described previously (62) and were conducted in accordance with the ENCODE guidelines. Briefly, approximately 1 × 10^7^ cells were used for one IP. Cells were fixed in 1% formaldehyde for 10 min at room temperature with swirling. Crosslinking was stopped by adding glycine to a final concentration of 125 mM and washing twice in ice-cold PBS. The crosslinked mESCs were harvested by scraping, pelleted, and resuspended in 1 ml of lysis buffer containing 50 mM Tris–HCl, pH 8.0, 10 mM EDTA, 1% SDS, and cOmplete EDTA-free protease inhibitor cocktail (Roche, Merck). Chromatin was sonicated using the Bioruptor sonicator for 2 to 10 cycles of 30 s ON and 30 s OFF at “high” power, in order to shear DNA to an average fragment size of 200 to 500 bp. Cell extract was diluted 10-fold in ChIP dilution buffer (20 mM Tris–HCl, pH 8.0, 2 mM EDTA, 1% Triton X-100, 150 mM NaCl, cOmplete EDTA-free). One hundred fifty microliters of anti-mouse IgG Dynabeads (Invitrogen, Thermo Fisher Scientific) for mouse antibody or 75 μl of Protein A Dynabeads (Invitrogen, Thermo Fisher Scientific) for rabbit antibody was preincubated with 10 μg of antibody at 4 °C overnight; chromatin was then added and incubated at 4 °C for at least another 6 h. The beads were then washed five times in radioimmunoprecipitation assay buffer (50 mM Hepes–KOH, pH 7.0, 0.5 M LiCl, 1 mM EDTA, 0.7% deoxycholate, and 1% NP-40) and once in TE buffer (10 mM Tris–HCl, pH 8.0, 1 mM EDTA). Immunoprecipitated samples were eluted and reverse crosslinked by incubation in elution buffer (50 mM Tris–HCl, pH 8.0, 10 mM EDTA, and 1% SDS) at 65 °C overnight. Genomic DNA was then extracted with a QIAquick PCR purification kit (Qiagen).

ChIP DNA was subjected to high-throughput sequencing analysis (ChIP-seq). The libraries were constructed using Ion Xpress Plus Fragment Library Kit (Thermo Fisher Scientific), as described previously (62). Adaptor-ligated samples were amplified by 15 cycles of PCR and purified by E-Gel SizeSelect (Thermo Fisher Scientific). The libraries were sequenced on the Ion Proton sequencer (Thermo Fisher Scientific). Both reference genomes and gene models were obtained from Illumina iGenome website (https://support.illumina.com/sequencing/sequencing_software/igenome.html). All ChIP-seq datasets were trimmed down to 50 bp using FASTP (version 0.23.2) (65) and were aligned to the mouse reference genome GRCm38/mm10 with Bowtie 2 (version 2.2.5) (66) with default parameters. Peaks were called using MACS3 (model-based analysis of ChIP-seq) (version 3.0.0a6) (67) with the following parameters: "-g mm --broad-cutoff 0.1 --broad" for H3K4me1/broad peaks, "-g mm --q 0.05" for H3K4me2/narrow peaks, and "-g mm --q 0.01" for DNA-binding TFs/narrow peaks such as SMAD1/5 and KDM1A. Peak calling for H3K9me2 was performed using Epic2 (version 0.0.52) (68) with the following parameters: "-gn mm10 -fs 50 -bin 500 -g 8 -kd -fdr 0.05." Common regions of the duplicate ChIP-seq data were generated using R package ChIPpeakAnno (version 3.36.1) (69). Assignment of genes to regions was done using the ChIPpeakAnno, taking the closest gene within a maximum distance of 10 kb.

Bigwig files with 1-bp resolution were generated and scaled to 1× coverage (reads per genome coverage, 1× RPGC) using bamCoverage function of deeptools2 package (version 3.5.1) (70) with the following parameters: "--binSize 1 --normalizeUsing RPGC --extendReads 200 --ignoreDuplicates --effectiveGenomeSize 2308125349." Input tracks were thereafter subtracted by using deeptools2 bigWigCompare with the following parameters: "--binSize 1 --operation subtract." Regions with value less than 0 were removed using bwtool (version 1.0) (71) with the following parameters: "remove less 0." ChIP-seq tracks showing peak distribution on the genome were visualized with pyGenomeTracks (version 3.7) from deeptools (72). Both bigWig (signal) and BED (peak regions) files were plotted, and replicates were overlaid for direct comparison. Tracks were aligned to the mm10 reference genome and displayed at locus-level resolution. Figure parameters, including genomic coordinates, *y*-axis scaling, scale bars (*e*.*g*., 5 kb scale), and gene annotations, were formatted for clear interpretation. The genomic regions were annotated with ChIPpeakAnno to nearest protein-coding genes. Over-representation analysis of GO terms were conducted with enrichGO function of clusterProfiler package (version 4.10.1) (73).

### ChIP-Atlas database analysis

To obtain a clue, we analyzed ChIP-seq data available through ChIP-Atlas database (35, 36). The data were downloaded on November 5, 2024. First, The Enrichment Analysis tool of TF revealed that 1027 ChIP-seq datasets out of total 7312 datasets (mm10, Experiment type: ChIP-TFs and others, Cell type class: PSC) overlapped more than or equal to 20 gene loci in those of 40 DEGs. Furthermore, the colocalization analysis showed that 482 datasets, or 135 proteins, colocalized with SMAD1 on a genome-wide scale (mm10, Antigen: Smad1, Cell type class: PSC).

### Transcriptome analysis: microarray and RNA-seq

Publicly available microarray datasets were obtained from Gene Expression Omnibus (GEO) using the R package GEOquery (74) and DEGs were analyzed using the R package DESeq2 (75). Publicly available RNA-seq datasets were obtained from GEO. The raw FASTQ files were adaptor trimmed and quality controlled by FASTP (version 0.23.2) (65). For datasets with larger size, 50 million reads were randomly picked using seqtk software (https://github.com/lh3/seqtk). The FASTQ files were aligned to the mouse reference genome GRCm38/mm10 with Bowtie2 (version 2.2.5) (66) and quantified with RSEM (version 1.2.28) (76) with the default parameter. After quantification, genes were analyzed for differential expression using the R package DESeq2 (75), and *p* values obtained by two-sided Wald's test and corrected for multiple testing using the Benjamini and Hochberg method.

### Gene set enrichment analysis

For microarray/RNA-seq data with two or more replicates, the list of genes was sorted based on stat variable from the DESeq2 (version 1.26.0). For RNA-seq data without replicates (n = 1), the list of genes was sorted based on log2 fold change. Preranked gene set enrichment analysis with a custom gene set comprising the S15down genes (20 genes) was performed using R package fgsea (version 1.30.0) (77) with 10,000 permutations.

### Single-cell RNA-seq analysis

The cells were gently digested into single cells using 0.05% trypsin–EDTA (Gibco) and were resuspended at ∼1000 cells/μl. Single-cell RNA-seq libraries were generated from parental E14 mouse ESCs (WT) and *Smad1*^−/−^;*Smad5*^−/−^ double KO cells (S1/5 dKO) using the Chromium Single Cell 3′ Reagent Kit v3 chemistry (10X Genomics) with a target of 3000 cells per sample, according to the manufacturer's protocol. The libraries were sequenced on the Illumina HiSeq X 10 platform in paired-end read mode with 150 bp per read, which was conducted by GENEWIZ from Azenta Life Sciences. Raw sequencing data were processed using Cell Ranger software (version 3.0.2) with mm10 reference genome (provided by 10X Genomics). Single-cell RNA-seq data of day 6 EB generated from *Kdm1a*-deficient mESCs (n = 2 for each group) (29) was retrieved from the GEO under the accession number GSE232255.

The 10X CellRanger output data files (barcodes.tsv, features.tsv, and matrix.tsv) were analyzed using the R package Seurat (version 4.3.0.1) (78). Reads were processed by counting unique molecular identifiers. Cells with fewer than 1000 unique feature counts and more than 5% mitochondrial RNA counts were excluded to remove low-quality cells. Single-cell gene expression counts were log-normalized using the NormalizeData function, with a scale factor of 10,000. The top 2000 variable genes were identified using the “vst” method in the FindVariableFeatures function. The normalized data were scaled using the ScaleData function and integrated (or batch-corrected) using the FindIntegrationAnchors and IntegrateData functions. Graph-based clustering was then performed on the integrated data using the FindClusters function at a resolution of 0.5, and cluster identities were manually annotated based on marker gene expression. Clusters were extracted using the subset function. Gene expression levels were represented as violin plots using VlnPlot function or as feature plots using FeaturePlot function. Pseudo-bulk analysis of scRNA-seq was performed using the AverageExpression function in Seurat. In our case, we had only n = 1 for each condition, so pseudo-bulk analysis was performed without biological replicates. To score each cell's position in the cell cycle, we used tricycle (version 1.4.0) (32), a R package that estimates the cell cycle position based on the abundance of known cell-cycle genes (*e*.*g*., *Top2a* and *Smc4*).

### Flow cytometry and fluorescence-activated cell sorting

Flow cytometry analyses and fluorescence-activated cell sorting were performed on Cell Sorter SH800S (Sony) as described (79). To establish E14-6×BRE(GC)-mAG; MERVL-tdTomato reporter cells, LV-6×BRE(GC)-mAG-Neo-infected E14 cells were established and mAG^high^ cells were sorted, followed by infection of LV-MERVL-tdTomato-Hyg and tdTomato^high^ cells were sorted. E14-6×BRE(GC)-mAG;MERVL-tdTomato reporter cells were treated with or without 100 ng/ml BMP4 or 10 μM of DMH1 for 24 h.

Cells were gently digested into single cells using 0.05% trypsin–EDTA (Gibco) and were filtered through a 40 μm cell strainer. The samples were analyzed on Cell Sorter SH800S. Data analysis was performed using Cell Sorter Software (version 2.1.5) (Sony) and visualized using R package ggplot2. Results showed a similar tendency in more than three independent experiments. Representative data are shown in Figure S2*G*.

### IP/protein pulldown assay

HEK293T cells were transiently transfected using Lipofectamine 2000 and incubated for 36 h before analysis. Cells were lysed in a lysis buffer containing 20 mM Hepes, 100 mM NaCl, 0.5 mM EDTA, 10% glycerol, 0.2% NP-40, and cOmplete EDTA-free protease inhibitor cocktail (cOmplete, EDTA free; Roche Applied Science). FLAG-tagged protein was immunoprecipitated using anti-mouse IgG Dynabeads (Invitrogen, Thermo Fisher Scientific) and anti-FLAG M2 antibody (F3165; Sigma–Aldrich, Merck). The beads (30 μl) were washed once in ice-cold PBS, resuspended in PBS, and preincubated with 2.5 μg of antibody on a rotating wheel at 4 °C overnight, followed by two washes with cold PBS. Cells were lysed in the lysis buffer and incubated with the pretreated Dynabeads for 4 h. Proteins in immunoprecipitates or cleared cell lysates were subjected to SDS-PAGE. Western blot analysis was performed using the indicated antibodies.

To detect endogenous protein interactions, approximately 1 × 10^6^ of mESCs were used for each condition. Thirty microliters of anti-mouse IgG Dynabeads were washed once in ice-cold PBS, resuspended in PBS, and preincubated with 2.5 μg of anti-SMAD1 antibody (913C1b; Bio Matrix Research) on a rotating wheel at 4 °C overnight, followed by two washes with cold PBS. Cells were lysed in the lysis buffer and incubated with the pretreated Dynabeads for 4 h. Proteins in immunoprecipitates or cleared cell lysates were subjected to SDS-PAGE. Western blot analysis was performed using the indicated antibodies.

Protein pulldown assay was performed using anti-mouse IgG Dynabeads and anti-SMAD1 antibody (913C1b; Bio Matrix Research). Thirty microliters of beads was washed once in ice-cold PBS, resuspended in PBS, and preincubated with 2.5 μg of antibody on a rotating wheel at 4 °C overnight, followed by two washes with cold PBS. The pretreated Dynabeads were washed once in 200 μl of PBS with Tween-20 (PBST) and incubated with recombinant proteins (500 ng each) as indicated in 200 μl of PBST at room temperature for 15 min. The beads were then washed three times in 200 μl of PBST and eluted with SDS-PAGE sample buffer and subjected to SDS-PAGE. Western blot analysis was performed using the indicated antibodies.

### Western blot analysis

Western blot analysis was performed essentially as described previously (62). Samples were separated by SDS-PAGE, blotted onto a polyvinylidene difluoride membrane (Pall), and the chemiluminescence signal was detected by a luminescence image analyzer (LAS-4000; Fujifilm). All primary antibodies for Western blotting were used at a 1:1000 dilution. Horseradish peroxidase–conjugated secondary antibodies were used at a 1:10,000 dilution. The intensities of the bands were quantified using ImageJ software (80). Uncropped Western blot images are presented in Figure S13.

### Materials and correspondence

Materials used or generated in this study will be available upon reasonable request, and a material transfer agreement may be required. Further information and requests for resources and reagents should be directed to and will be fulfilled by the corresponding author, Masato Morikawa (morikawa-tky@umin.ac.jp).

### Statistical information

No statistical method was used to predetermine sample size, no data were excluded from the analyses, and the investigators were not blinded to allocation during experiments and outcome assessment. All statistical tests and number of observations are stated in figure panels or legends. Unless otherwise indicated, statistical analyses were carried out using R. Data distribution was assumed to be normal, but this was not formally tested. Data are presented as scatter plots with bar graphs, which indicate mean ± SD. A two-sided Welch's *t* test, an unequal variances *t* test, was used to compare the two conditions. Differences between the conditions were analyzed by Tukey's honest significant difference test corrected for multiple comparisons. In the half-violin plots of scRNA-seq analysis, each cell was treated as an independent replicate, and differences in gene expression between conditions were analyzed using the Wilcoxon rank sum test.

## Data availability

• All newly generated ChIP-seq and single-cell RNA-seq data were deposited at the GEO under accession numbers GSE268911 and GSE268912. All other data supporting the findings of this study are available from the corresponding author on reasonable request.
• This article does not report original code.
• Any additional information required to reanalyze the data reported in this work article is available from the corresponding author upon request.

Publicly available datasets used in this study were obtained from GEO with the following accession number.

• ChIP-seq

Smad1/5 ChIP-seq in mESCs: GSE70579 (GSM1810977, GSM1810978, and GSM1810980)

Smad4 ChIP-seq in mESCs: GSE125116 (GSM3563725, GSM3563728)

Smad1/5 ChIP-seq in mEpiLCs: GSE70579 (GSM1810982, GSM1810984, and GSM1810986)

KDM1A ChIP-seq in mouse epiblast stem cells: DRP001313 (DRX013314, DRX013325, and DRX013326)

H3K4me2 ChIP-seq in mEpiLCs: GSE155089 (GSM4694570, GSM4694721, and GSM4694727)

• Microarray

*Kdm1a* KO mESCs: GSE21131 (GSM528560, GSM528561, GSM528562, GSM528563, GSM528564, and GSM528565)

• RNA-seq

*Kdm1a* KO mESCs: GSE232255 (GSM7324597, GSM7324599, GSM7324600, GSM7324601, GSM7324602, and GSM7324603)

*Dnmt1/3a/3b* triple KO mESCs: GSE64910 (GSM1583042, GSM1583043, GSM1583052, and GSM1583053)

*Zfp57* KO mESCs: GSE31182 (GSM773068, GSM773069)

*Trim28* KO mESCs: GSE74278 (GSM1916163, GSM1916164, GSM1916165, GSM1916166, GSM1916167, and GSM1916168)

*Kdm2b* (Fbxl10) KO mESCs: GSE65583 (GSM1600473, GSM1600474)

*Kmt2b* (MLL2) KO mESCs: GSE98988 (GSM2629687, GSM2629688, GSM2779052, and GSM2779053)

*Kdm6a* (UTX) KO mESCs: GSE97703 (GSM2575700, GSM2575701, GSM2575702, GSM2575703, GSM2575704, and GSM2575705)

*Tet1/2/3* triple KO mESCs: GSE56986 (GSM1372648, GSM1372650)

*Ing5* KO mESCs: GSE196946 (GSM5905508, GSM5905509, GSM5905514, and GSM5905515)

*Smarca4* (BRD1) knockdown mESCs: GSE57170 (GSM1376650, GSM1376651, GSM1376652, and GSM1376653)

*Brd9* knockdown mESCs: GSE111264 (GSM3027471, GSM3027472, GSM3027473, and GSM3027474)

• scRNA-seq

GSE232255 (GSM7324710, GSM7324711, GSM7324712, and GSM7324713).

## Supporting information

This article contains supporting information (23, 29, 34, 62, 81, 82).

## Conflict of interest

The authors declare that they have no conflicts of interest with the contents of this article.
