# Supplementary material for: SMAD1/5-mediated recruitment of the histone demethylase KDM1A controls cell fate programs in embryonic stem cells
Source: J Biol Chem. 2025 Aug 12;301(9):110591. doi: 10.1016/j.jbc.2025.110591 (PMC12450650; doi:10.1016/j.jbc.2025.110591)
Supplement: Supplementary Information [file mmc2.pdf]

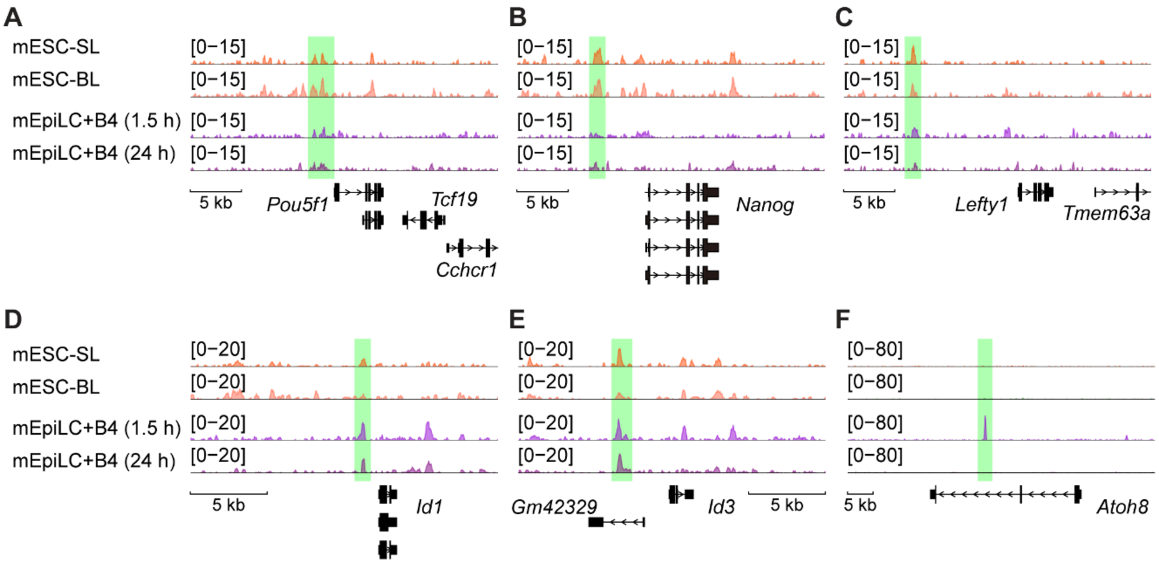

Figure S1. SMAD1/5 binding regions in naïve ESCs and formative/primed EpiLCs.

(**A−F**) Genome browser tracks of the indicated normalized ChIP-seq signals at the indicated gene loci of BMP-SMAD1/5 target genes and control genes of WT and S1/5 dKO cells. The read counts were first normalized to 1× genome coverage (reads per genome coverage, RPGC) and then normalized to input. (A,B) *Pou5f1* (which encodes OCT4): a marker gene of pluripotent stem cells. *Nanog*: a marker gene of naïve pluripotency. In the naïve condition, SMAD1/5 locate at the OCT4-SOX2-NANOG (OSN) target sites such as the enhancer regions of *Pou5f1* and *Nanog* (34), which is in accordance with the fact that SMAD1/5 make complex with NANOG (81) and KLF4 (23). In the formative/primed condition, however, SMAD1/5 binding to the −5 kb *Nanog* enhancer was decreased, possibly because one of the naïve-specific marker genes *Nanog* is not expressed in the formative condition while OCT4 is expressed in the formative/primed conditions. (C) *Lefty1*: one of the marker genes of the formative/primed conditions. SMAD1/5 commonly bind the same regions in both conditions, possibly because chromatin is open in naïve condition although *Lefty1* is not expressed. (D,E) *Id1* and *Id3*: well-established target genes

of the BMP-SMAD signaling pathway. SMAD1/5 commonly bind the same regions in both conditions. (F) *Atoh8*: a SMAD1/5 target gene which restrains cellular plasticity (62, 82). SMAD1/5 bind an enhancer region of the gene only in BMP4-treated cells in the formative condition.

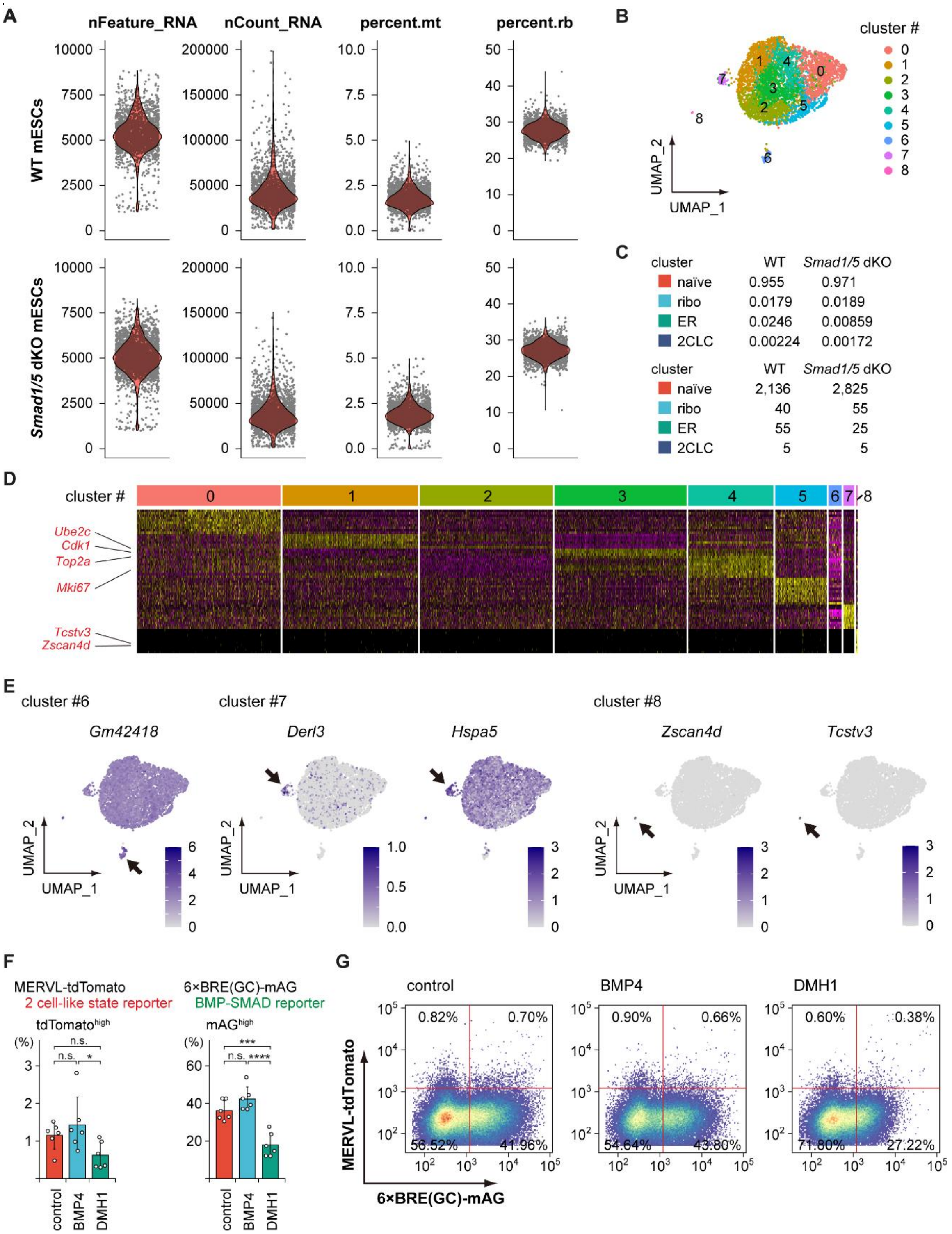
A
nFeature_RNA
nCount_RNA
percent.mt
percent.rb
WT mESCs
Smad1/5 dKO mESCs
B
cluster #
0
1
2
3
4
5
6
7
8
UMAP_1
UMAP_2
C
cluster WT Smad1/5 dKO
naïve 0.955 0.971
ribo 0.0179 0.0189
ER 0.0246 0.00859
2CLC 0.00224 0.00172
cluster WT Smad1/5 dKO
naïve 2,136 2,825
ribo 40 55
ER 55 25
2CLC 5 5
D
cluster #
Ube2c
Cdk1
Top2a
Mki67
Tcstv3
Zscan4d
E
cluster #6
Gm42418
cluster #7
Derl3
Hspa5
cluster #8
Zscan4d
Tcstv3
F
MERVL-tdTomato
2 cell-like state reporter
tdTomatohigh
6×BRE(GC)-mAG
BMP-SMAD reporter
mAGhigh
(%)
n.s.
control
BMP4
DMH1
G
control
BMP4
DMH1
0.82%
0.70%
56.52%
41.96%
0.90%
0.66%
54.64%
43.80%
0.60%
0.38%
71.80%
27.22%
MERVL-tdTomato
6×BRE(GC)-mAG

Figure S2. Single-cell RNA-seq analysis of *Smad1/5*-deficient mESCs and parental mESCs.

(**A**) Quality control (QC) metrics for genes, reads, mitochondrial, and ribosomal transcripts per cell as determined using Seurat pre-processing violin plots. The violin plots illustrate the number of genes detected per cell (nFeature_RNA), the number of reads per cell (nCount_RNA), the percent of mitochondrial transcripts per cell (percent.mt), and the percent of ribosomal transcripts per cell (percent.rb).

(**B**) Uniform manifold approximation and projection (UMAP) visualization of the transcriptional heterogeneity of parental E14 mESCs (WT, n = 2,236) and *Smad1/5*-deficient mESCs (S1/5 dKO, n = 2,910). Cells are colored based on the clusters they belong to.

(**C**) The tables summarize the percentages (top) and raw cell counts (bottom) of each population in WT and S1/5 dKO samples. The corresponding bar plot is shown in Figure 1F.

(**D**) Heatmaps representing top 10 differentially expressed genes for each cluster of the integrated data of WT and S1/5 dKO cells. Color represents the mRNA expression levels, which are scaled by z-transformation and limited to a minimum scale from −2.5 to 2.5 (purple to yellow).

(**E**) Uniform manifold approximation and projection (UMAP) plots showing the mRNA expressions of the selected marker genes of the cluster #6, #7 or #8 in the integrated data of WT and S1/5 dKO cells. Each arrow indicates the indicated cluster.

(**F,G**) Flow cytometry analysis of the E14-6×BRE(GC)-monomeric Azami-Green (mAG);MERVL-tdTomato reporter cells with/without BMP4 or DMH1. Since serum

contains BMP ligands, a condition supplemented with the BMP receptor kinase inhibitor DMH1 is considered a BMP-negative condition. (G) Representative data of n = 6 independent experiments.

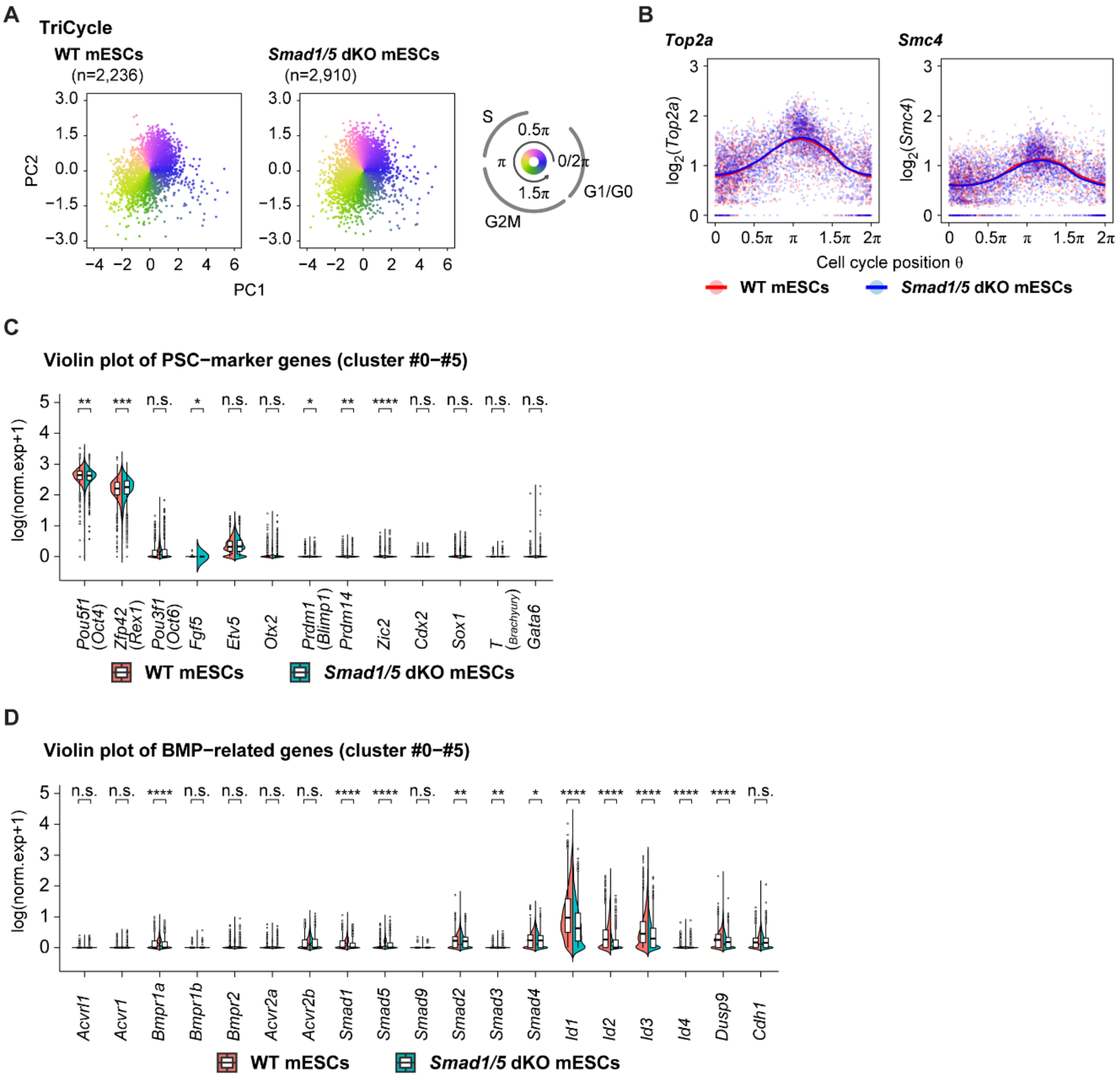

Figure S3. Single-cell RNA-seq comparison between Smad1/5-deficient and parental mESCs.

(**A,B**) Tricycle analysis to estimate cell cycle position. (A) UMAP plot of WT or S1/5 dKO cells representing the expression of cell cycle genes. 0.5 π indicates initial S phase, π indicates initial G2M phase, 1.5 π indicates middle of M phase and 1.75–0.25 π indicates G1/G0 state. (B) The expression dynamics of *Top2a* and *Smc4* using the inferred cell-cycle

position, with a periodic loess line.

(**C,D**) Half-violin plots showing the distribution of the mRNA expression levels of BMP-related genes in WT and S1/5 dKO cells within cluster #0−#5. Each cell was treated as an independent replicate, and differences in gene expression between conditions were analyzed using the Wilcoxon rank sum test. (C) PSC marker genes. *Pou5f1*/OCT4 mark PSCs; *Zfp42* marks naïve PSCs; *Pou3f1*/OCT6, *Fgf5*, *Etv5*, and *Otx2* mark formative/primed PSCs; *Prdm1*, *Prdm14* mark PGC; *Zic2*, *Cdx2* mark trophoblast stem cells (TSCs); *Sox1*, *T*/Brachyury, and *Gata6* display lineage commitment. (D) BMP-related genes.

*, **, ***, ****, and n.s. represent $P < 0.05$, 0.01, 0.001, 0.0001, and not significant, respectively.

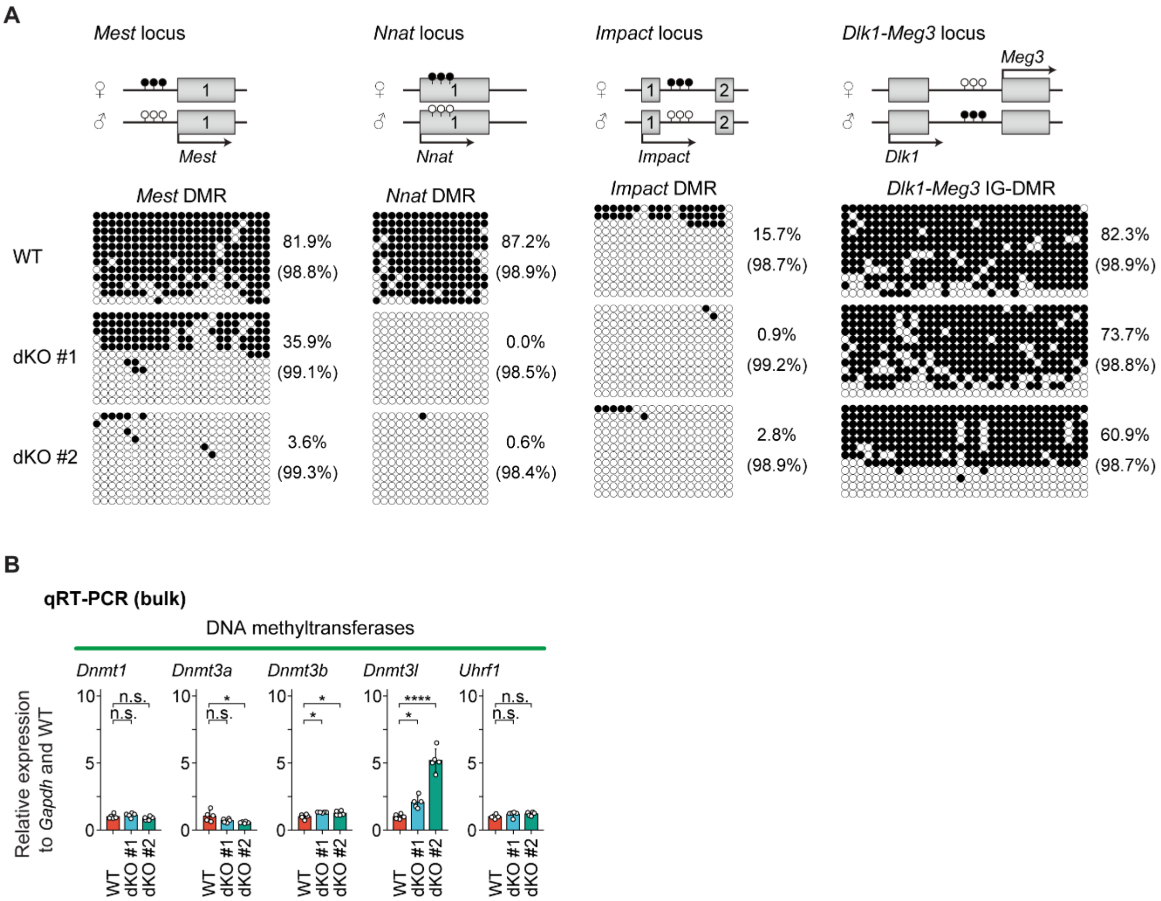

Figure S4. Bisulfite sequencing analysis of the SMAD1/5 binding regions in naïve ESCs.

(**A**) Bisulfite sequencing analysis of 5mC of differentially methylated regions (DMRs) of the SMAD1/5-repressed genes.

(**B**) qRT-PCR analysis of mRNA of indicated genes in WT and S1/5 dKO cells. *Gapdh* was used as endogenous control. Results of n = 5 independent experiments are presented as scatter plots with bar graphs which indicate mean ± SD. Differences between the conditions were analyzed by Tukey's HSD test corrected for multiple comparisons.

*, ****, and n.s. represent $P < 0.05$, 0.0001, and not significant, respectively.

A

ChIP-Atlas/Enrichment Analysis

Genome assembly: mm10
Antigen_class: TFs and others
Cell_class: Pluripotent stem cell

Overlap with gene loci of 40 DEGs

	≥20 gene loci	Total
# of dataset:	1,027	7,312
# of protein:	139	448

ChIP-Atlas/Colocalization

Genome assembly: mm10
Antigen: Smad1
Cell_class: Pluripotent stem cell

CoLo scores (genome-wide colocalization)

	≥5	Total
# of dataset:	482	4,286
# of protein:	135	348

B

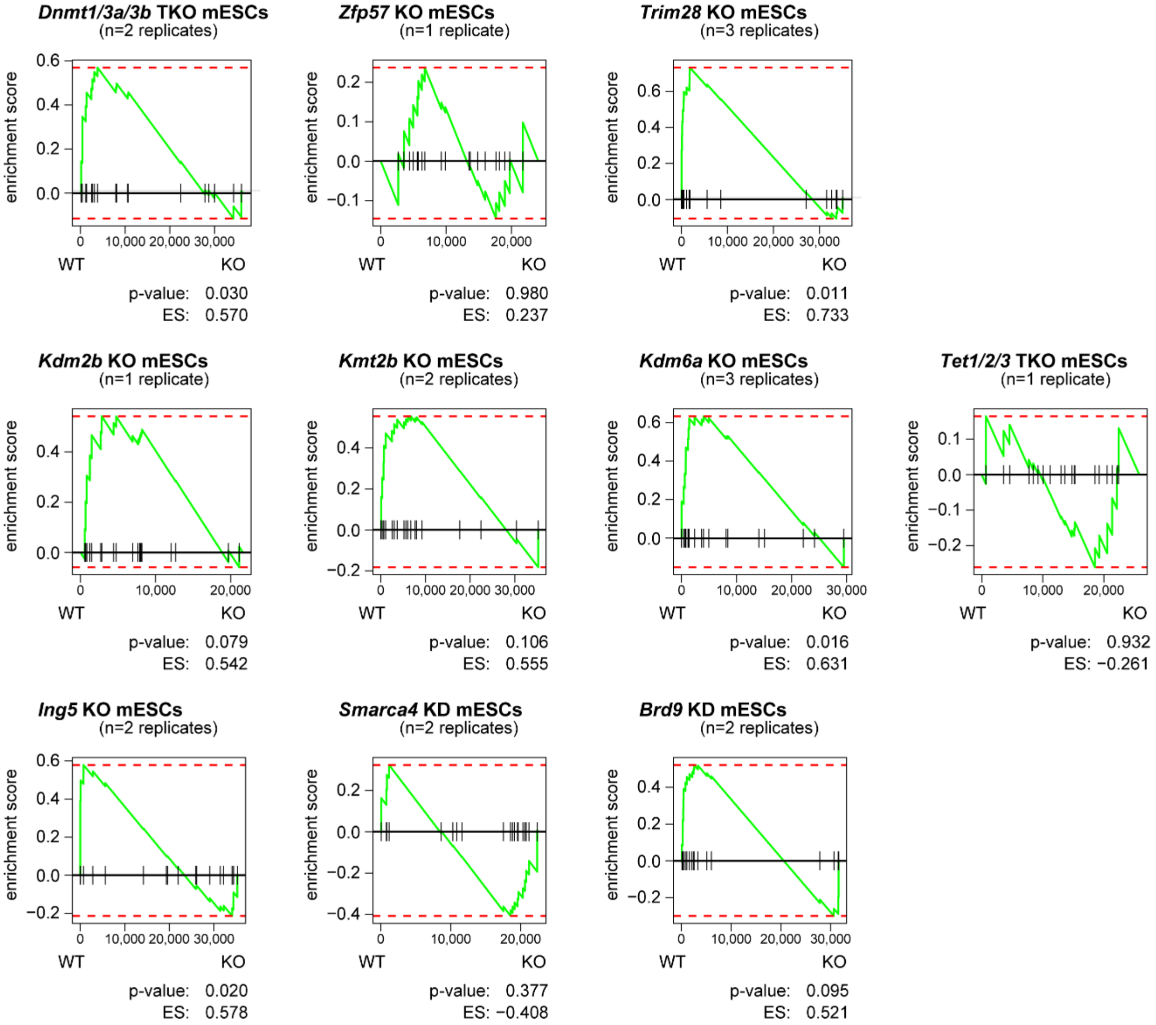

Figure S5. Involvement of KDM1A/LSD1 in regulation of the genes down-regulated by SMAD1/5.

(**A**) Tables provide detailed overview of the results obtained from ChIP-Atlas. Related to Figure 3B.

(**B**) GSEA plots showing the enrichment results of the custom gene set of the genes down-regulated by SMAD1/5 (S15down genes, 20 genes) in mESCs deficient in indicated gene(s) compared with control cells. ES: enrichment score. Related to Figure 3D.

```
                           268
Homo sapiens SMAD1         PKHWCSIVYYELNNRVGEAFHASSTS--VLVDGFTDPSNNKNRFCLGLLSNVNRNSTIEN
Homo sapiens SMAD5         PKHWCSIVYYELNNRVGEAFHASSTS--VLVDGFTDPSNNKSRFCLGLLSNVNRNSTIEN
Homo sapiens SMAD8         PQHWCSVAYYELNNRVGETFQASSRS--VLIDGFTDPSNNRNRFCLGLLSNVNRNSTIEN
Homo sapiens SMAD2         PAFWCSIAYYELNQRVGETFHASQPS--LTVDGFTDPSNSE-RFCLGLLSNVNRNATVEM
Homo sapiens SMAD3         PAFWCSISYYELNQRVGETFHASQPS--MTVDGFTDPSNSE-RFCLGLLSNVNRNAAVEL
Homo sapiens SMAD4         PEYWCSIAYFEMDVQVGETFKVPSSCPIVTVDGYVDPSGG-DRFCLGQLSNVHRTEAIER
Mus musculus SMAD1         PKHWCSIVYYELNNRVGEAFHASSTS--VLVDGFTDPSNNKNRFCLGLLSNVNRNSTIEN
Mus musculus SMAD5         PKHWCSIVYYELNNRVGEAFHASSTS--VLVDGFTDPSNNKSRFCLGLLSNVNRNSTIEN
Mus musculus SMAD8         PQHWCSVAYYELNNRVGETFQASSRS--VLIDGFTDPSNNRNRFCLGLLSNVNRNSTIEN
Mus musculus SMAD2         PAFWCSIAYYELNQRVGETFHASQPS--LTVDGFTDPSNSE-RFCLGLLSNVNRNATVEM
Mus musculus SMAD3         PAFWCSISYYELNQRVGETFHASQPS--MTVDGFTDPSNSE-RFCLGLLSNVNRNAAVEL
Mus musculus SMAD4         PEYWCSIAYFEMDVQVGETFKVPSSCPVVTVDGYVDPSGG-DRFCLGQLSNVHRTEAIER
D. melanogaster MAD        PAFWASIAYYELNCRVGEVFHCNNNS--VIVDGFTNPSNNSDRCCLGQLSNVNRNSTIEN

                           326
Homo sapiens SMAD1         TRRHIGKGVHLYYVG-GEVYAECLSDSSIFVQSRNCNYHHGFHP-TTVCKIPSGCSLKIF
Homo sapiens SMAD5         TRRHIGKGVHLYYVG-GEVYAECLSDSSIFVQSRNCNFHHGFHP-TTVCKIPSSCSLKIF
Homo sapiens SMAD8         TRRHIGKGVHLYYVG-GEVYAECVSDSSIFVQSRNCNYQHGFHP-ATVCKIPSGCSLKVF
Homo sapiens SMAD2         TRRHIGRGVRLYYIG-GEVFAECLSDSAIFVQSPNCNQRYGWHP-ATVCKIPPGCNLKIF
Homo sapiens SMAD3         TRRHIGRGVRLYYIG-GEVFAECLSDSAIFVQSPNCNQRYGWHP-ATVCKIPPGCNLKIF
Homo sapiens SMAD4         ARLHIGKGVQLECKGEGDVWVRCLSDHAVFVQSYYLDREAGRAPGDAVHKIYPSAYIKVF
Mus musculus SMAD1         TRRHIGKGVHLYYVG-GEVYAECLSDSSIFVQSRNCNYHHGFHP-TTVCKIPSGCSLKIF
Mus musculus SMAD5         TRRHIGKGVHLYYVG-GEVYAECLSDSSIFVQSRNCNFHHGFHP-TTVCKIPSSCSLKIF
Mus musculus SMAD8         TRRHIGKGVHLYYVG-GEVYAECVSDSSIFVQSRNCNYQHGFHP-ATVCKIPSGCSLKVF
Mus musculus SMAD2         TRRHIGRGVRLYYIG-GEVFAECLSDSAIFVQSPNCNQRYGWHP-ATVCKIPPGCNLKIF
Mus musculus SMAD3         TRRHIGRGVRLYYIG-GEVFAECLSDSAIFVQSPNCNQRYGWHP-ATVCKIPPGCNLKIF
Mus musculus SMAD4         ARLHIGKGVQLECKGEGDVWVRCLSDHAVFVQSYYLDREAGRAPGDAVHKIYPSAYIKVF
D. melanogaster MAD        TRRHIGKGVHLYYVT-GEVYAECLSDSAIFVQSRNCNYHHGFHP-STVCKIPPGCSLKIF

                           384
Homo sapiens SMAD1         NNQEFAQLLAQSVNHGFET----------------------------------VYELTKM
Homo sapiens SMAD5         NNQEFAQLLAQSVNHGFEA----------------------------------VYELTKM
Homo sapiens SMAD8         NNQLFAQLLAQSVHHGFEV----------------------------------VYELTKM
Homo sapiens SMAD2         NNQEFAALLAQSVNQGFEA----------------------------------VYQLTRM
Homo sapiens SMAD3         NNQEFAALLAQSVNQGFEA----------------------------------VYQLTRM
Homo sapiens SMAD4         DLRQCHRQMQQQAATAQAAAAAQAAAVAGNIPGPGSVGGIAPAISLSAAAGIGVDDLRRL
Mus musculus SMAD1         NNQEFAQLLAQSVNHGFET----------------------------------VYELTKM
Mus musculus SMAD5         NNQEFAQLLAQSVNHGFEA----------------------------------VYELTKM
Mus musculus SMAD8         NNQLFAQLLAQSVHHGFEV----------------------------------VYELTKM
Mus musculus SMAD2         NNQEFAALLAQSVNQGFEA----------------------------------VYQLTRM
Mus musculus SMAD3         NNQEFAALLAQSVNQGFEA----------------------------------VYQLTRM
Mus musculus SMAD4         DLRQCHRQMQQQAATAQAAAAAQAAAVAGNIPGPGSVGGIAPAISLSAAAGIGVDDLRRL
D. melanogaster MAD        NNQEFAQLLSQSVNNGFEA----------------------------------VYELTKM

                           410
Homo sapiens SMAD1         CTIRMSFVKGWGAEYHRQDVTSTPCWIEIHLHGPLQWLDKVLTQMGSPHNPISSVS
Homo sapiens SMAD5         CTIRMSFVKGWGAEYHRQDVTSTPCWIEIHLHGPLQWLDKVLTQMGSPLNPISSVS
Homo sapiens SMAD8         CTIRMSFVKGWGAEYHRQDVTSTPCWIEIHLHGPLQWLDKVLTQMGSPHNPISSVS
Homo sapiens SMAD2         CTIRMSFVKGWGAEYRRQTVTSTPCWIELHLNGPLQWLDKVLTQMGSPSVRCSSMS
Homo sapiens SMAD3         CTIRMSFVKGWGAEYRRQTVTSTPCWIELHLNGPLQWLDKVLTQMGSPSIRCSSVS
Homo sapiens SMAD4         CILRMSFVKGWGPDYPRQSIKETPCWIEIHLHRALQLLDEVLHTMPIADPQPLD
Mus musculus SMAD1         CTIRMSFVKGWGAEYHRQDVTSTPCWIEIHLHGPLQWLDKVLTQMGSPHNPISSVS
Mus musculus SMAD5         CTIRMSFVKGWGAEYHRQDVTSTPCWIEIHLHGPLQWLDKVLTQMGSPLNPISSVS
Mus musculus SMAD8         CTIRMSFVKGWGAEYHRQDVTSTPCWIEIHLHGPLQWLDKVLTQMGSPHNPISSVS
Mus musculus SMAD2         CTIRMSFVKGWGAEYRRQTVTSTPCWIELHLNGPLQWLDKVLTQMGSPSVRCSSMS
Mus musculus SMAD3         CTIRMSFVKGWGAEYRRQTVTSTPCWIELHLNGPLQWLDKVLTQMGSPSIRCSSVS
Mus musculus SMAD4         CILRMSFVKGWGPDYPRQSIKETPCWIEIHLHRALQLLDEVLHTMPIADPQPLD
D. melanogaster MAD        CTIRMSFVKGWGAEYHRQDVTSTPCWIEIHLHGPLQWLDKVLTQMGSPHNAISSVS
```

Figure S6. Sequence conservation of the MH2 domains of SMAD proteins.

Multiple amino acid sequence alignment of the MH2 domain of SMAD protein of different species. Colored blocks indicate identical (orange) or highly conserved (yellow) amino acids compared to human SMAD1 (orange).

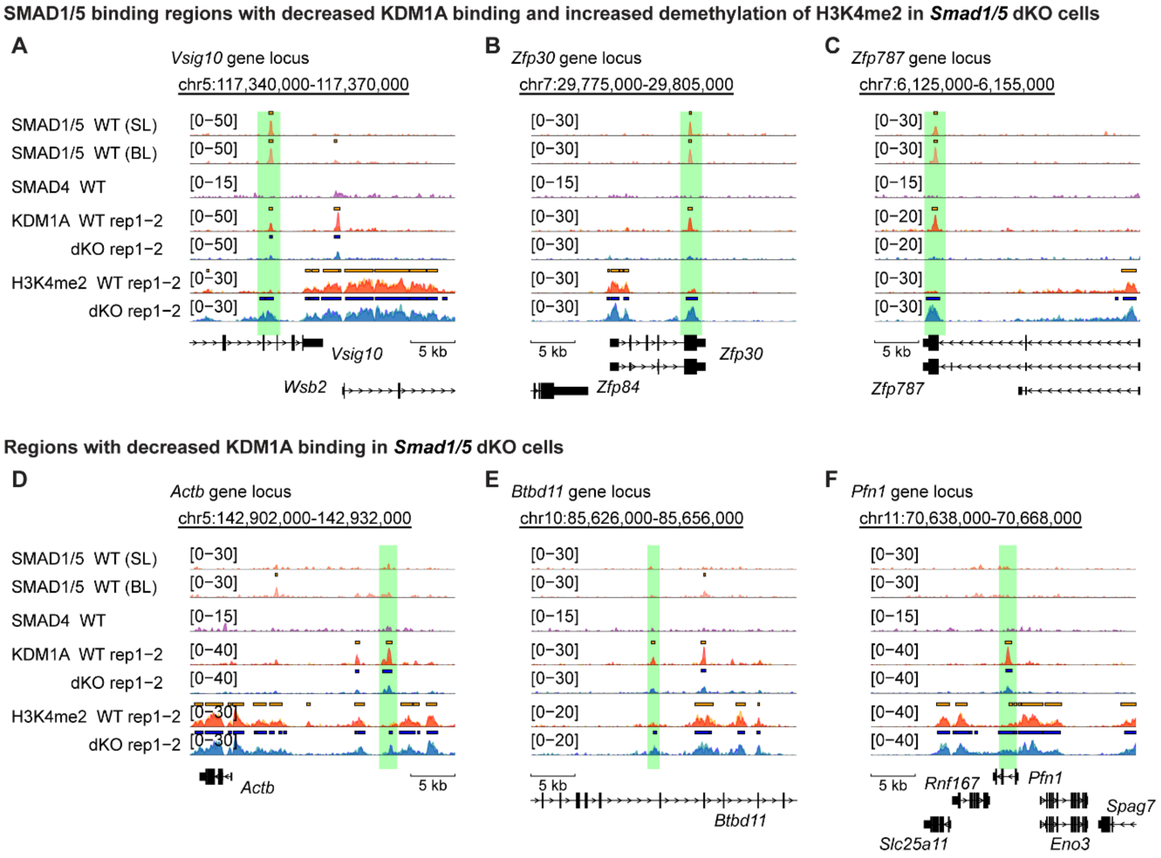

Figure S7. Gene loci with decreased KDM1A binding and increased demethylation of H3K4me2 in S1/5 dKO cells.

(**A−F**) Genome browser tracks of the indicated normalized ChIP-seq signals at the gene loci of indicated genes of WT and S1/5 dKO cells. The read counts were first normalized to 1× genome coverage (reads per genome coverage, RPGC) and then normalized to input. Each ChIP-seq data is presented in a different color. Solid bars above ChIP peaks represent called peaks. (A−C) Examples of SMAD1/5 binding regions with decreased KDM1A binding and increased demethylation of H3K4me2 in S1/5 dKO cells. (D−F) Examples of SMAD1/5-unbound regions with decreased KDM1A binding and increased demethylation of H3K4me2 in S1/5 dKO cells.

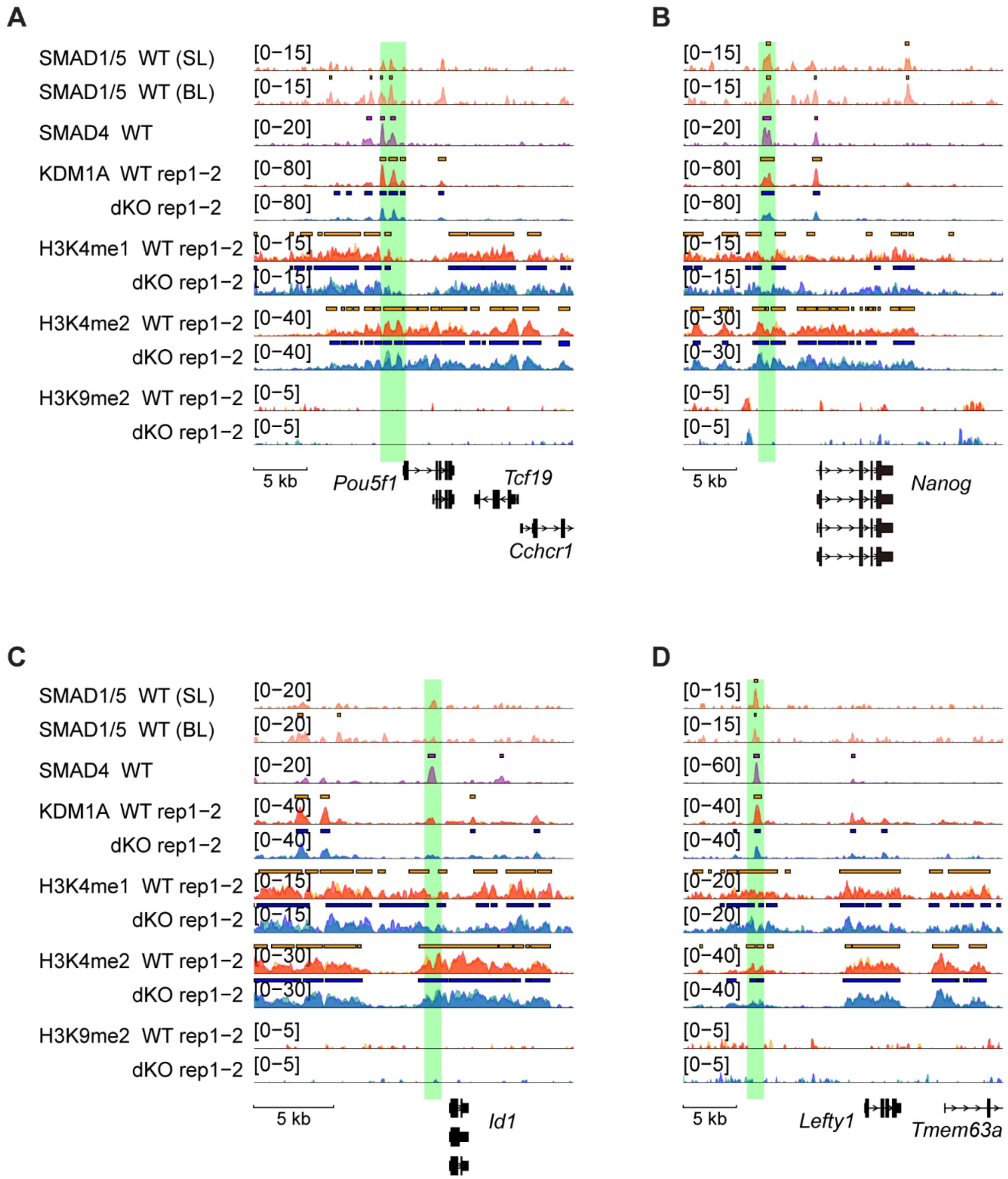

Figure S8. H3K4me2 and KDM1A enrichment in the SMAD1/5-SMAD4 binding regions in naïve ESCs.

(**A−D**) Genome browser tracks of the indicated normalized ChIP-seq signals at the gene loci of indicated genes of WT and S1/5 dKO cells. The read counts were first normalized

to 1× genome coverage (reads per genome coverage, RPGC) and then normalized to input. Each ChIP-seq data is presented in a different color. Solid bars above ChIP peaks represent called peaks.

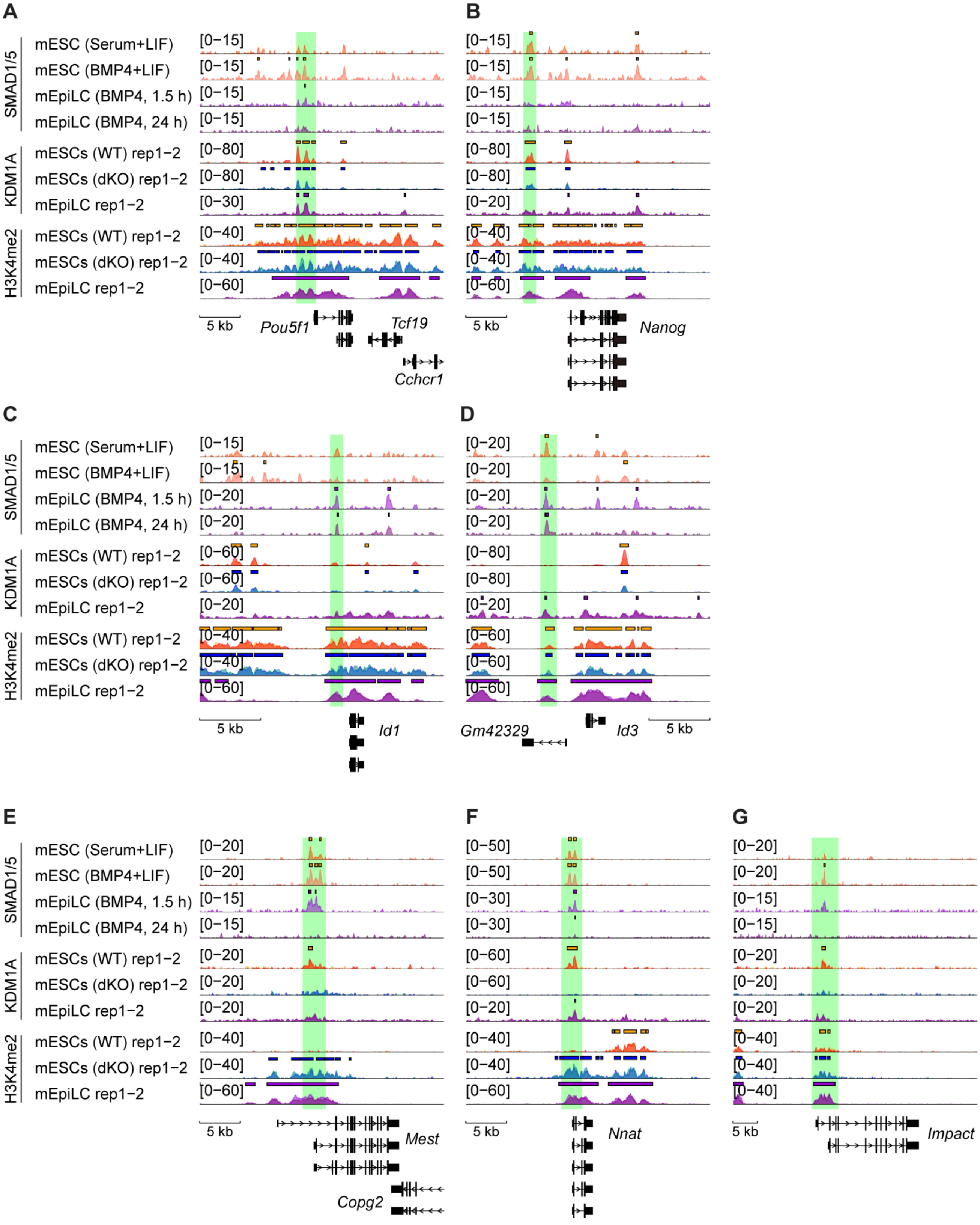
A
B
C
D
E
F
G
SMAD1/5
KDM1A
H3K4me2
mESC (Serum+LIF)
mESC (BMP4+LIF)
mEpiLC (BMP4, 1.5 h)
mEpiLC (BMP4, 24 h)
mESCs (WT) rep1−2
mESCs (dKO) rep1−2
mEpiLC rep1−2
5 kb
Pou5f1
Tcf19
Cchcr1
Nanog
Id1
Gm42329
Id3
Mest
Copg2
Nnat
Impact

Figure S9. Evaluation of SMAD1/5, KDM1A, and H3K4me2 at SMAD1/5-bound regions in naïve mESCs and formative/primed EpiLCs.

(**A−G**) Genome browser tracks showing normalized ChIP-seq signals for the indicated factors at selected gene loci in WT and Smad1/5 dKO mESCs, and WT mEpiLCs. Read counts were first normalized to 1× genome coverage (reads per genome coverage, RPGC) and then to input. Each ChIP-seq dataset is shown in a different color. Solid bars above ChIP peaks indicate peak-calling results. Previously reported SMAD1/5 peaks in mEpiLCs at 1.5 and 24 hours after BMP4 treatment are annotated. *Pou5f1* (Oct4) is bound by SMAD1/5 in both naïve and formative/primed cells, while *Nanog* is bound only in the naïve state. *Id1* and *Id3* serve as known SMAD1/5-SMAD4 target genes.

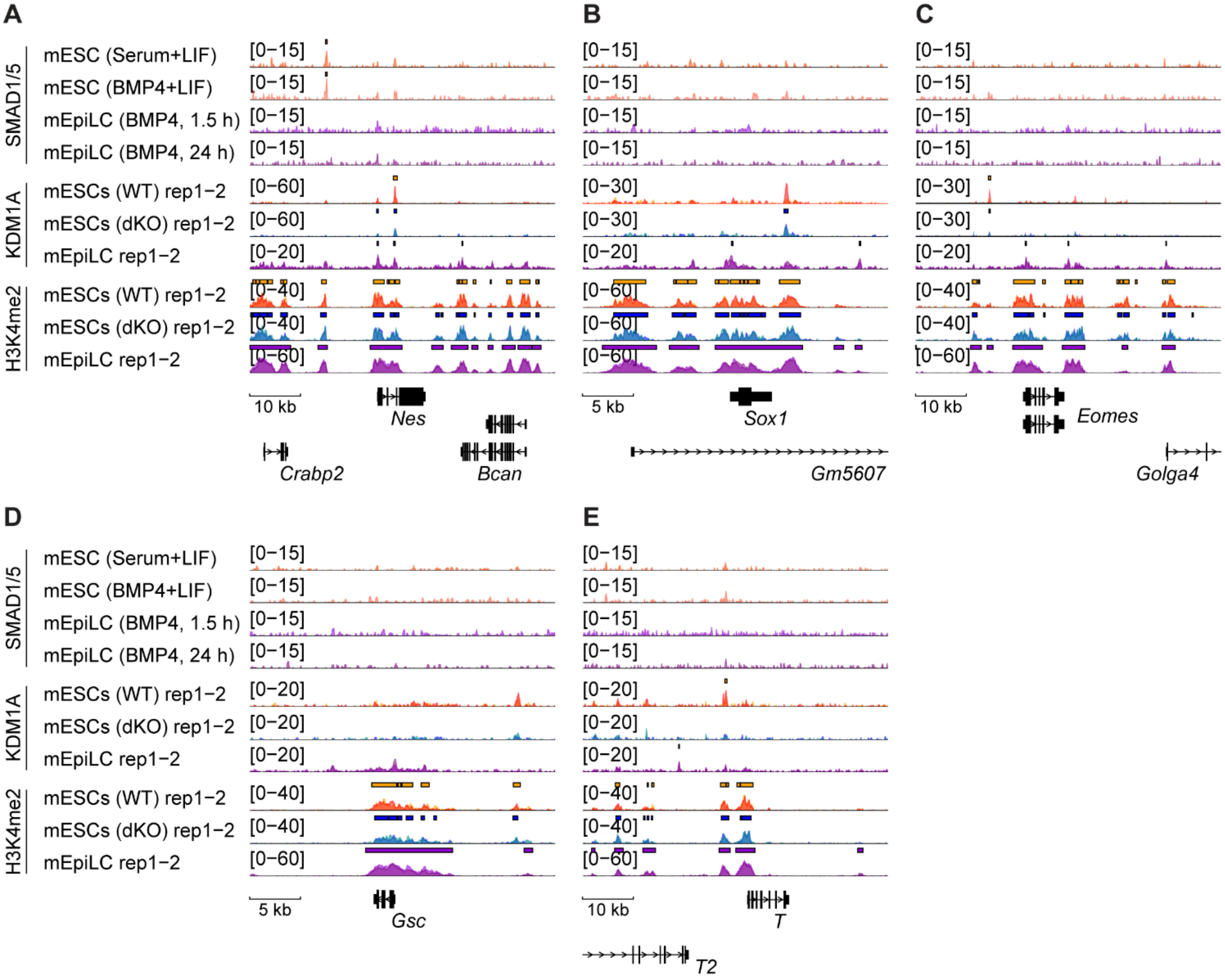

Figure S10. Evaluation of SMAD1/5, KDM1A, and H3K4me2 at SMAD1/5-bound regions in naïve mESCs and formative/primed EpiLCs.

(**A−E**) Genome browser tracks showing normalized ChIP-seq signals for the indicated factors at the loci of genes associated with differentiation. Read counts were first normalized to 1× genome coverage (reads per genome coverage, RPGC), and then to input. Each ChIP-seq dataset is displayed in a different color. Solid bars above ChIP peaks indicate called peaks. For SMAD1/5 ChIP-seq data in mEpiLCs, previously identified peaks are marked (224 peaks at 1.5 hours and 132 peaks at 24 hours after BMP4 treatment).

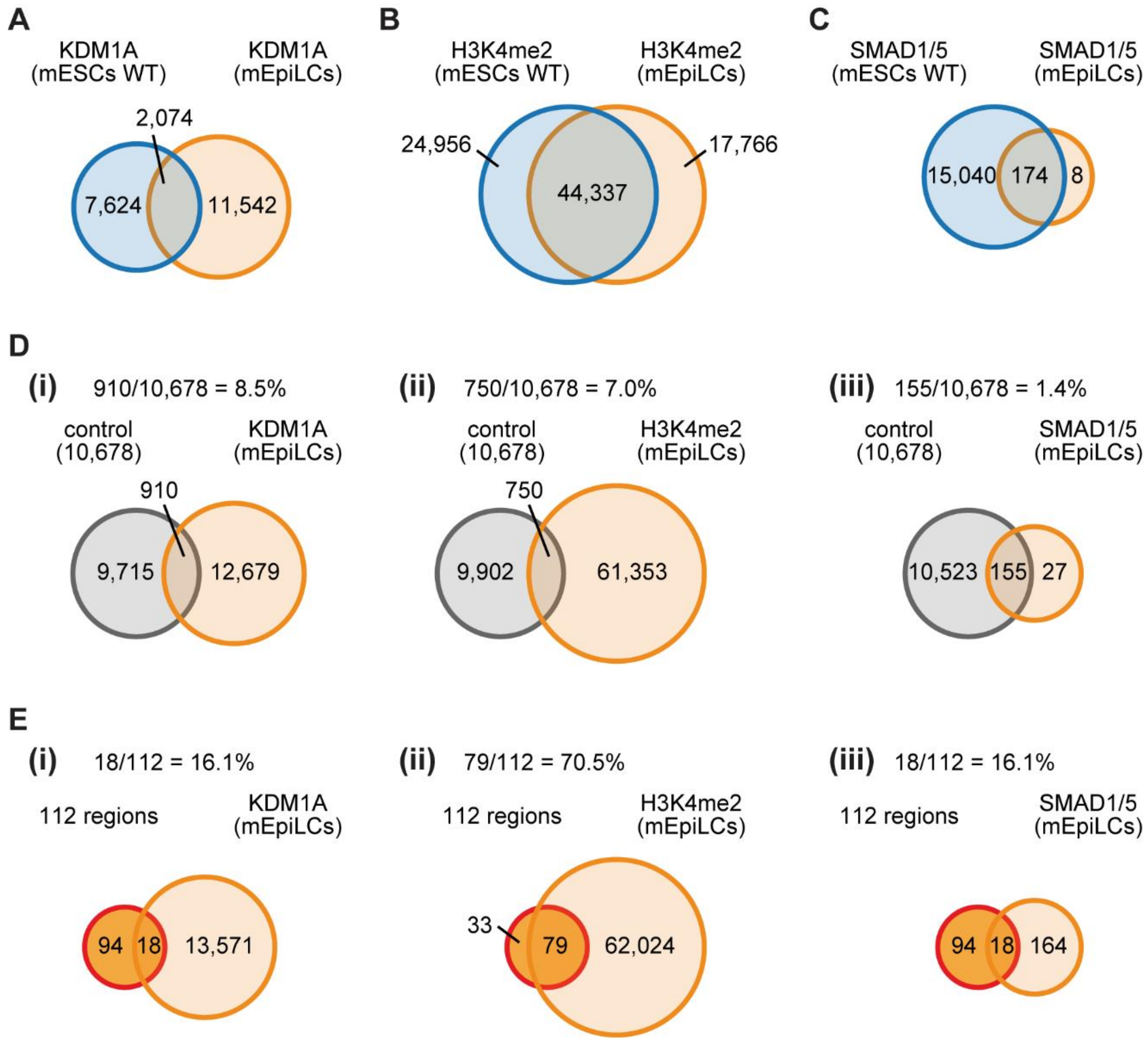

Figure S11. Evaluation of SMAD1/5, KDM1A, and H3K4me2 at SMAD1/5-bound regions in naïve mESCs and formative/primed EpiLCs.

(**A−E**) Venn diagrams indicating the overlap of genomic regions identified using ChIP-seq data. (A–C) Overlaps of regions bound by KDM1A, H3K4me2, and SMAD1/5 in WT mESCs or WT mEpiLCs. (D) Control regions that are SMAD1/5-positive but H3K4me2-negative in mESCs (n = 10,678), and their overlap with the indicated ChIP-seq peaks in mEpiLCs. (E) The 112 genomic regions that represent candidate loci where SMAD1/5

recruits KDM1A to demethylate H3K4me2 (as defined in Figures 6B and 6C), and their overlap with the indicated ChIP-seq peaks in mEpiLCs.

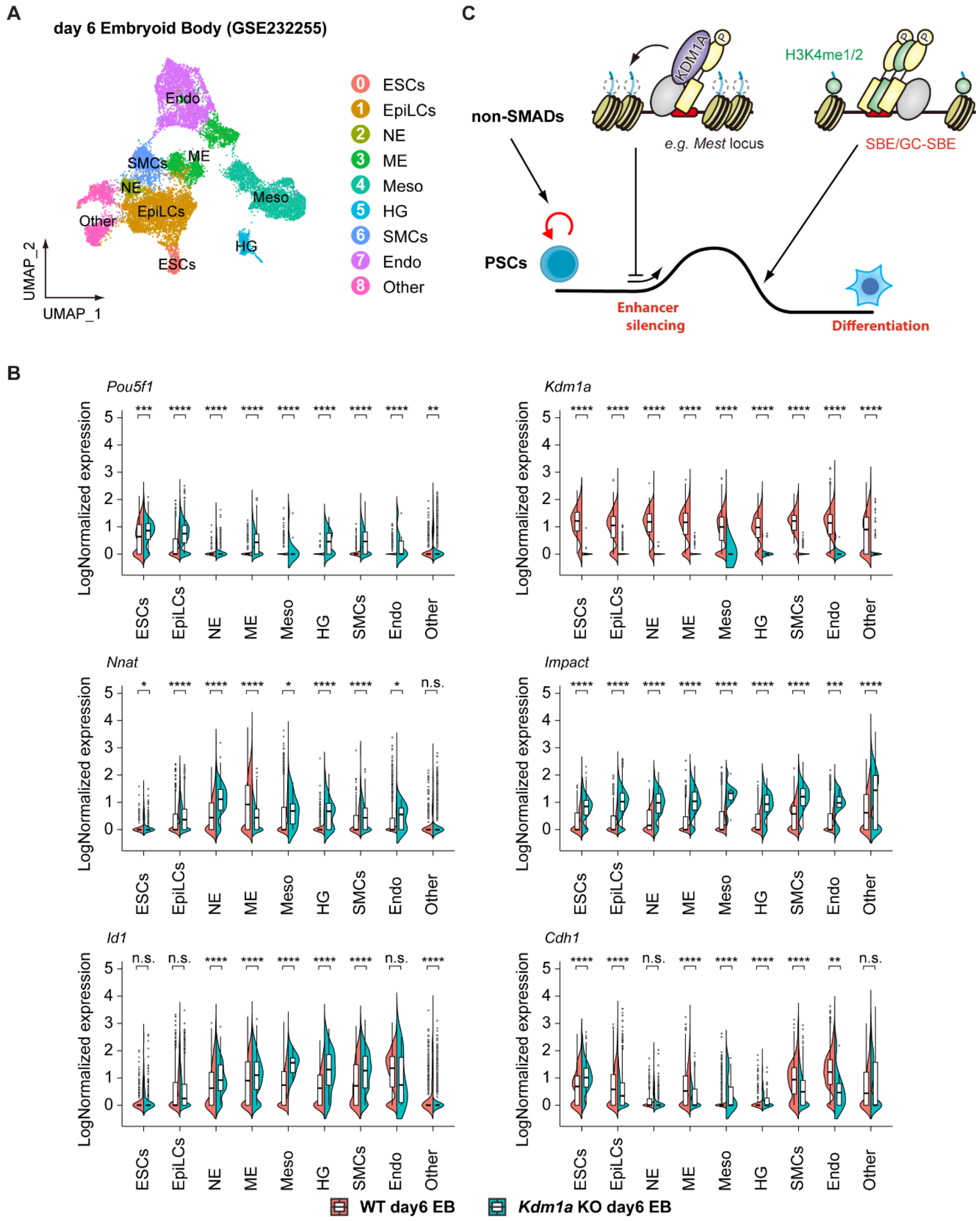

Figure S12. Effects of KDM1A loss in PSC differentiation to specific lineages.

(**A**) UMAP plot showing the cell types present in the day 6 embryoid body generated from *Kdm1a*-deficient mESCs (n=7,623) and control (n=10,214) (29). Cells are colored based

on the clusters they belong to. NE: Sox1$^+$ neuroectoderm progenitor; NM: Nascent Mesoderm; ME: Mesendoderm; Meso: Mesoderm; HG: Flk1$^+$ hemangioblast; SMC: Smooth Muscle Cells; Endo: Endoderm; VE: Visceral Endoderm.

(**B**) Half-violin plots showing the distribution of the mRNA expression levels of the indicated genes in either WT or *Kdm1a*-deficient cells within the indicated subpopulations. Each cell was treated as an independent replicate, and differences in gene expression between conditions were analyzed using the Wilcoxon rank sum test.

(**C**) Model for roles of BMP signaling in mESCs. The BMP-SMAD pathway is dispensable to maintain mESCs, while the SMAD complex with SMAD4 directs differentiation towards specific lineages, such as mesoderm. Our findings demonstrate that the SMAD1/5-KDM1A complex represses genes related to developmental process toward several lineages, including neuroectoderm, in naïve mESCs. SBE: SMAD binding element; GC-SBE: GC-rich SBE.

Figure 4A

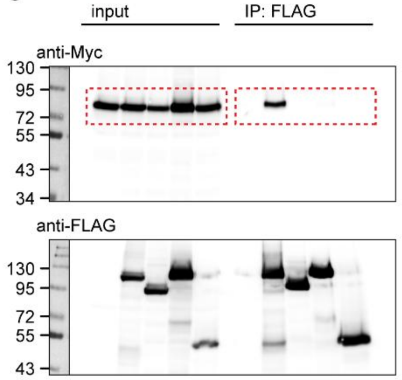

Figure 4B

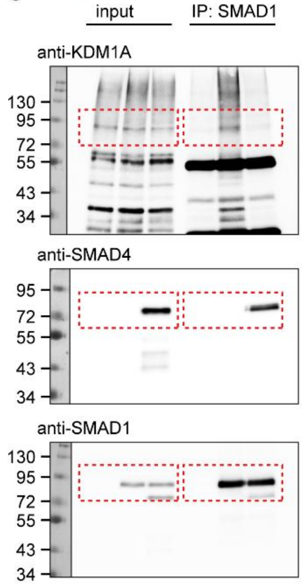

Figure 4C

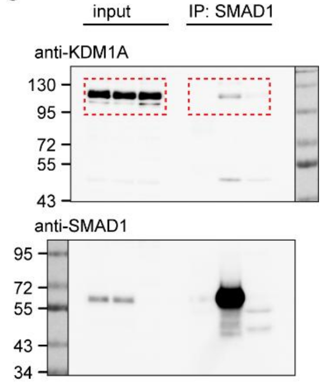

Figure 4F

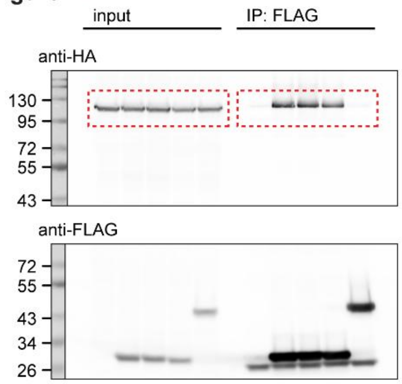

Figure 4G

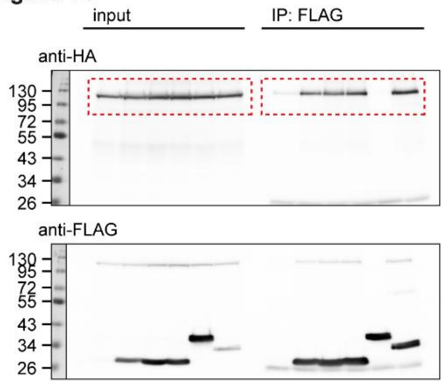

Figure 4H

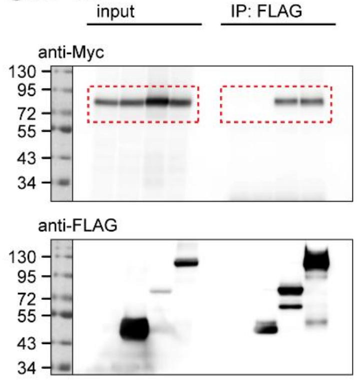

Figure 4I

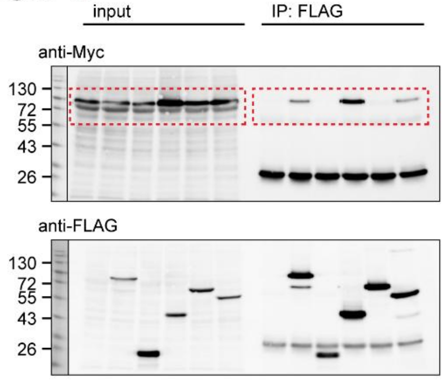

Figure S13. Uncropped Western blot images.

Uncropped images of all Western blots are shown, as well as the approximate extent of the cropped region.
